# Assessing the impact of the 2018 Changchun Changsheng vaccine incident on childhood vaccination in China

**DOI:** 10.1038/s43856-023-00339-0

**Published:** 2023-08-22

**Authors:** Zhiyuan Hou, Xiaozhen Lai, Yang Liu, Mark Jit, Heidi J. Larson, Hai Fang

**Affiliations:** 1 School of Public Health, NHC Key Laboratory of Health Technology Assessment, Fudan University, Shanghai, China; 2 Department of Health Policy and Management, School of Public Health, Peking University, Beijing, China; 3 Health Economics Research Centre, Nuffield Department of Population Health, University of Oxford, Oxford, UK; 4 Department of Infectious Disease Epidemiology, Faculty of Epidemiology and Population Health, London School of Hygiene and Tropical Medicine, London, UK; 5 Centre for Mathematical Modelling of Infectious Diseases, London School of Hygiene and Tropical Medicine, London, UK; 6 School of Public Health, University of Hong Kong, Hong Kong SAR, China; 7 China Center for Health Development Studies, Peking University, Beijing, China; 8 Peking University Health Science Center-Chinese Center for Disease Control and Prevention Joint Research Center for Vaccine Economics, Peking University, Beijing, China

**Keywords:** Public health, Preventive medicine, Vaccines

## Abstract

**Background:**

The 2018 Changchun Changsheng vaccine incident is an emergent public health event in China with reports of DTaP vaccines with compromised efficacy. This study aimed to estimate the impact of the vaccine incident on real-world vaccination behaviors in China.

**Methods:**

A cross-sectional survey was conducted in ten provinces in 2019. Vaccination records were collected from 5294 children aged 6-59 months, with information on 75,579 vaccine doses for seven National Immunization Program (NIP) vaccines and two non-NIP vaccines received from 2014 to 2019. Chi-square test, interrupted time series, and logistic regression were used to evaluate the impacts of vaccine incident on vaccination delay, measured as the proportion of delayed doses out of total doses in schedule.

**Results:**

Here we show significant increases in doses delayed ≤ 3 months (19.12% to 22.51%, *p* = 0.000) and > 3 months (7.17% to 11.82%, *p* = 0.000) for DTaP vaccine after the incident. By scaling nationally, there will be extra 2.1 million doses delayed ≤ 3 months and 2.8 million doses delayed > 3 months in the year following this incident. More guardians choose expensive private-market substitutes containing DTaP elements over government-funded DTaP vaccines. Controlling for socio-demographic factors, doses scheduled after the incident have higher odds of delays for DTaP vaccine (OR: 3.49, 95% CI: 3.08–3.96) and other NIP vaccines (OR: 2.76, 95% CI: 2.55–2.99), but not for non-NIP vaccines.

**Conclusions:**

The observed delays in the incident-involved DTaP vaccine immunization reflect the negative effects of the vaccine incident on vaccination behaviors under the NIP. However, its effects seem minimal for non-NIP vaccines.

## Plain Language Summary

In the 2018 Changchun Changsheng vaccine incident, the vaccine manufacturer was accused of producing substandard vaccines with compromised efficacy. This study assessed the impacts of the vaccine incident on subsequent vaccination of children in China. We found that vaccinations occurred later than normal for the specific vaccine involved in the incident, but there was no impact on the timing of other childhood vaccines. Children’s guardians also chose different vaccines from that involved in the vaccine incident. Information about how children’s guardians reacted to this incident could be used to improve the information provided in future about childhood vaccination, and be used to decide how vaccination programs could respond following similar incidents.

## Abstract (web summary)

Hou, Lai et al. estimate the impact of reports regarding a childhood vaccine with compromised efficacy on subsequent vaccination behaviors. The administration of the reported vaccine was delayed, while there was minimal effect on other vaccinations.

## Introduction

Receiving recommended childhood vaccinations on schedule is a cornerstone of public health, but incidents involving vaccine safety concerns affect public confidence in and timely uptake of vaccines^1^. In the age of social media, misinformation around vaccine safety and anti-vaccine movements have proliferated^2^, and vaccine hesitancy has been considered one of the top ten threats to global health by the World Health Organization^3^. Vaccine incidents worldwide, such as the misreporting of the association between measles-mumps-rubella (MMR) vaccine and autism^4^ and the suspected safety concerns around human papillomavirus vaccines in Japan^5^, have contributed to this growing global health threat. Maintaining public confidence and acceptance of vaccines has become increasingly important as timely vaccination is a major defense against new and emerging disease outbreaks, such as the COVID-19 pandemic^6^.

China has experienced a series of serious vaccine incidents over the last decade that have significantly undermined public confidence in vaccines^7,8^. The most recent and widespread one is the Changchun Changsheng vaccine incident, as a major public health event with extensive social influence. In July 2018, Changchun Changsheng Biotechnology Company was charged by China Food and Drug Administration (CFDA) with two counts of malpractice: (i) manufacturing and selling substandard diphtheria-tetanus-acellular-pertussis (DTaP) vaccines (November 2017), and (ii) illegal production of freeze-dried rabies vaccines by making up production and inspection records (July 2018)^9^. As one of these contraventions, the titer indicators of the sample-test DTaP vaccines produced by Changchun Changsheng Biotechnology Company were detected to be substandard based on the requirements of the CFDA, resulting in 247,359 substandard DTaP vaccines being administered to Chinese children^9^. The CFDA clarified that the affected vaccines were not fully efficacious, but met safety standards and would not directly harm children’s health. Although no long-term sequelae or deaths were officially reported as a result of these substandard vaccines, claims that the substandard vaccines were poisonous spread widely on the internet and social media sites. This incident evoked nationwide anxiety about the safety of vaccines and vaccine production in China^10^. After regulatory investigations, this company was fined $1.3 billion and declared bankrupt^11^. This incident culminated in the rapid enactment of the first Vaccine Administration Act in just one year^12^.

Although a series of cross-sectional surveys on vaccine confidence conducted following this vaccine incident reported a loss of public confidence^13–17^, none have drawn links from attitudes about vaccines to vaccination outcomes, such as vaccination delays or refusal. Some researchers believe that this incident was unlikely to have affected childhood vaccination rates due to the widespread support for the government-funded National Immunization Program (NIP) combined with strict vaccination requirements for school enrollment in China^18^. However, up to now there has been little evidence to substantiate this. There is also little literature to examine the effects of vaccine incidents on individuals’ vaccination behaviors across the world. Using data from a nationwide survey with childhood vaccination records, our study aims to evaluate the effect of the Changchun Changsheng vaccine incident on vaccination behaviors, especially focusing on the degree and duration of its effect and differential effects across vaccine types. This would provide evidence to fight potential vaccine incidents or crises in the future, particularly in the dual era of the COVID-19 pandemic and infodemic for mass vaccination, such as the administration of expired COVID-19 vaccines.

In this study, significant increases in delayed doses are observed following the vaccine incident for the DTaP vaccine and some other NIP vaccines, while no significant changes are observed for non-NIP vaccines. After the incident, more guardians choose the self-paid quadrivalent DTaP-Hib or pentavalent DTaP-IPV/Hib doses over government-funded DTaP vaccines involved in the incident.

## Methods

### Study design

We conducted a national cross-sectional survey from August to October 2019, around one year after the Changchun Changsheng vaccine incident, and collected children’s vaccination records till the survey time. A total of 148 vaccination centers (based in community health centers) from 10 provinces/provincial-level cities were approached to join an on-site survey on childhood vaccination records and household characteristics for children aged 6-59 months. This study was approved by the Peking University Institutional Review Board (IRB00001052-19076) and registered at clinicaltrials.gov (NCT04038333).

A pilot study was conducted for 30 guardians of children to engage in the design of the study and survey questionnaire in a non-study-site community. The formal survey adopted a multistage cluster sampling method, as extensively elaborated in Supplementary Note 1 and Supplementary Fig. 1^19,20^. First, 10 of 31 provinces/provincial-level cities were selected as our study sites in mainland China based on geographical location and financial governance division. Second, a capital city and a non-capital city were selected in each province (for each provincial-level city, an economically developed district and a less-developed district were selected). Third, two subdistricts/counties were chosen in each city or district. Fourth, in each subdistrict/county, three to four communities and the corresponding vaccination centers were sampled according to socio-economic strata. Fifth, guardians (parents or grandparents) of all children aged 6–59 months visiting the sampled vaccination centers on a given day during the survey period were invited to participate in the survey. In China, all children under five years of age are supposed to receive NIP vaccines based on the routine vaccination schedule at these vaccination centers. Thus, the on-site survey participants could be a representative sample of the local children, as the NIP vaccine coverage reached around 99% in China in 2019 according to official statistics^21^.

Guardians of children were interviewed by trained interviewers using an online questionnaire system on a portable Android device (PAD) which allowed quality control in a timely manner. The survey questionnaire covered the socio-demographic characteristics of the children (e.g., child’s age, gender, and number of children in a family) and their guardians (e.g. guardian’s age, relationship with the child, ethnicity, education level, household income, status of residence, and place of residence). Using PAD, we took snapshots of children’s vaccination records where the type and date of different vaccine doses received by each child were clearly written or printed^22^. Written informed consent was obtained from children’s guardians.

The sample size was initially set to 3840 (Supplementary Note 1), and was increased in practice to accommodate the response rate and ensure data integrity. In the survey, 6 668 children were recruited, among whom the guardians of 5384 (80.74%) children agreed to provide their vaccination records, and the records of 5294 (79.39%) were legible and complete with snapshots of every page.

### Outcome measures

Our study examined the vaccines scheduled for children below 18 months of age and widely used before the vaccine incident (Supplementary Table 1), including seven types of NIP vaccines which are delivered to children free of charge, and two types of non-NIP vaccines which are recommended but non-compulsory and self-paid. The NIP vaccines, all with national uptake around 99% prior to the vaccine incident, were DTaP vaccine (four doses), Bacillus Calmette-Guerin (BCG) vaccine (one dose), Hepatitis B (HepB) vaccine (three doses), Polio vaccine (three doses), MMR vaccine (one dose), Japanese encephalitis (JE) vaccine (one dose), and Hepatitis A (HepA) vaccine (one dose). Two widely used non-NIP vaccines of interest were Haemophilus influenza type b (Hib) conjugate vaccine (four doses) and Varicella vaccine (one dose), with national uptake around 40% and 70% before the vaccine incident, respectively^21,23^. Note that besides Hib and Varicella vaccines, we also collected information on other non-NIP vaccines available in the private market in China such as pneumococcal conjugate vaccine and rotavirus vaccine, but their uptake was too low to identify the impact of the vaccine incident^21^.

In our study, vaccine doses instead of vaccines were used as the analysis unit since the same child may experience doses before and after the incident for different types of vaccines and different doses of the same vaccine. For 5294 children with legible and complete vaccination records (out of 5384 children with available record books), a total of 75,579 vaccine doses received from 2014 to 2019 were reported for the nine types of vaccines mentioned above.

The outcome of interest was vaccination delay, and this outcome was compared before and after the vaccine incident. In this dose-based study, vaccination delay was measured as the proportion of delayed doses out of the total doses on schedule, and the delayed doses were defined as those not administered in the vaccination window. For the first dose of each vaccine (or only dose of a single-dose vaccine), the vaccination window was defined as the entire month of age at which the vaccine is scheduled (Supplementary Table 1), and for other doses, the vaccination window was defined as either the entire month of age at which the vaccine is scheduled or the entire month starting from a time point calculated by a fixed time interval (in months) between doses.

The duration of the vaccine incident’s effect on vaccination behaviors is our focus, and any dose delays of childhood DTaP vaccination may be important in the absence of a maternal pertussis vaccination program. According to the delay duration, vaccination delay can be divided into two categories: (i) short-term delay where vaccine dose is delayed but vaccinated within 3 months beyond the vaccination window, and (ii) long-term delay where vaccine dose is delayed for more than 3 months beyond the vaccination window^24^. As most vaccine doses are scheduled before six months of age with a one-month vaccination window, we set 3 months as the threshold of long-term delay, during which period the complete lack of vaccine protection would increase the risk of vaccine-preventable disease and disease outbreaks^24^. The long-term delay consists of two occasions, in which some doses were finally vaccinated after 3 months and the others have not been vaccinated until the survey time (Supplementary Table 2). Furthermore, to ensure comparability of data before and after the vaccine incident, doses within their vaccination window falling within three months before the survey time were excluded.

### Statistical analysis

Frequencies and proportions were used to describe the characteristics of children and their guardians. We conducted an interrupted time series analysis to examine the impact of the vaccine incident on vaccination delay across vaccine types, and its impact was modeled using a segmented generalized linear model (GLM) with pre-incident trends (104 weeks, July 17 2016–July 14 2018) and the weekly proportion of delayed doses in post-incident period (44 weeks, July 15 2018–May 19 2019)^25^. We then compared the total proportions of delayed doses before and after the vaccine incident using the chi-square test, and adopted an equal time period restricted to Jul 2017-May 2018 (before the incident) and Jul 2018-May 2019 (after the incident) as a sensitivity analysis to explore the robustness of the comparison.

Logistic regression was used to evaluate the association between vaccine incident and vaccination delays, and multinomial logistic regression was further adopted to investigate its associations with short-term and long-term delays separately. The dependent variable was whether one vaccine dose was delayed or not according to the vaccination schedule, and independent variables included whether the dose was scheduled after the vaccine incident, and the socio-demographic characteristics of children and their guardians.

In addition, both trivalent DTaP vaccine in NIP and quadrivalent DTaP-Hib or pentavalent DTaP-IPV/Hib vaccine outside the NIP are available for children in China. In the Changchun Changsheng vaccine incident, substandard vaccines were only reported for trivalent DTaP vaccines. Thus, guardians’ choice of different vaccines containing DTaP antigens was also examined to understand the change in vaccination behaviors after the vaccine incident. We then conducted additional analyses to explore the factors associated with the decision to procure self-paid vaccines.

Odds ratio (OR), relative-risk ratio (RRR), and their 95% confidence intervals (CIs) were presented. A two-sided *p*-value below 0.05 was considered statistically significant in the present study. All data were analyzed using Stata version 17.0 (Stata Corp., College Station, TX, USA)^26^.

### Reporting summary

Further information on research design is available in the Nature Portfolio Reporting Summary linked to this article.

## Results

Table 1 shows the socio-demographic characteristics of 5294 respondents and their children with complete and legible vaccination records. The distribution of surveyed children’s gender and age matched the target group consisting of children at immunized ages in China. Specifically, the proportion of boys surveyed slightly exceeded that of girls, with boys comprising 52.81% and girls 47.19% of the sample. For comparison, the China Population and Employment Statistics Yearbook 2019 reported a gender ratio of 53:47 (boys to girls) for children aged 0–5 years in 2018^27^. Additionally, the study identified four age categories, namely, < 1, 1-, 2-, and 3-5 years old, which accounted for 27.18%, 29.22%, 18.53%, and 25.07%, respectively. While the distribution of age groups was not fully consistent with the yearbook, this can be attributed to the fact that the targeted children of immunization ages tended to be younger (Supplementary Table 1).

**Table 1:** Characteristics of children and their guardians.

Characteristics	Number of observations	Proportion (%)
Total	5294	100.00%
Child’s age (years)		
< 1	1439	27.18%
1–2	1547	29.22%
2–3	981	18.53%
3–5	1327	25.07%
Child’s gender		
Female	2498	47.19%
Male	2796	52.81%
Number of children in a family		
1	2467	46.60%
≥ 2	2827	53.40%
Guardian’s age (years)		
< 30	1637	30.92%
30–39	2457	46.41%
40–49	458	8.65%
≥ 50	742	14.02%
Guardian’s relationship with the child		
Mother	3534	66.75%
Father	907	17.13%
Grandparent	853	16.11%
Ethnic groups		
Han	4984	94.14%
Ethnic minorities	310	5.86%
Guardian’s education level		
Elementary school or below	521	9.84%
Middle school	1355	25.60%
Senior high school or technical school	1194	22.55%
Three-year college or associate degree	997	18.83%
Bachelor’s degree or above	1227	23.18%
Quintiles of per capita monthly income		
Quintile 1 (CNY 0–1000)	1152	21.76%
Quintile 2 (CNY 1001–1600)	900	17.00%
Quintile 3 (CNY 1601–2400)	1063	20.08%
Quintile 4 (CNY 2401–3750)	1132	21.38%
Quintile 5 (CNY > 3751)	1047	19.78%
Status of residence		
Local resident	4027	76.07%
Inter-city migrant	1267	23.93%
Place of residence		
Rural	2139	40.40%
Urban	3155	59.60%
Province		
Shandong	522	9.86%
Beijing	551	10.41%
Chongqing	567	10.71%
Gansu	390	7.37%
Guangdong	511	9.65%
Henan	516	9.75%
Jiangxi	554	10.46%
Jilin	613	11.58%
Yunnan	545	10.29%
Shanghai	525	9.92%

*CNY* Chinese Yuan, 1 CNY = 0.14 USD in 2019.

### Comparison of vaccination delay before and after the vaccine incident

**Fig. 1:**
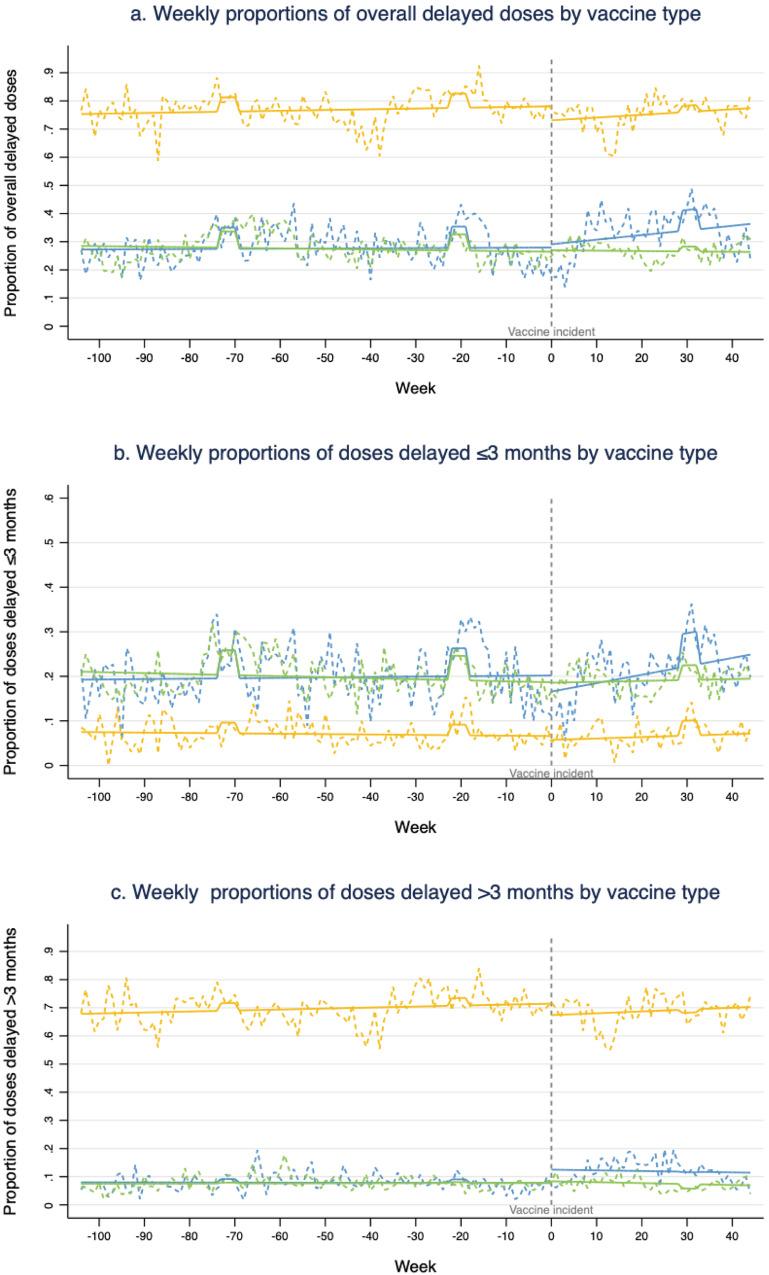
Weekly proportions of delayed doses by vaccine type before and after vaccine incident. **a**, **b**, **c** Respectively show the scenarios for overall delays, delays less than or equal to three months, and delays greater than three months. Data from 104 weeks before the vaccine incident to 44 weeks after the incident, with three lines in each figure representing DTaP doses (blue line), NIP doses excluding DTaP (green line), and non-NIP doses (orange line), respectively. Broken lines represent the observed proportions of delayed doses by week, and straight lines were fitted by a segmented generalized linear model (GLM) with pre-incident and post-incident trends. There are limited vaccination services available in Chinese New Year holidays: (1) weeks (−73, −70) for Chinese New Year in 2016; (2) weeks (−22, −19) for Chinese New Year in 2017; (3) weeks (29, 32) for Chinese New Year in 2018.

Figure 1 shows the impact of the vaccine incident on the weekly proportions of delayed vaccine doses estimated by interrupted time series analysis, and the numerical details of relative changes in levels and trends attributable to the vaccine incident are presented in Supplementary Table 3. For weekly proportions of delayed DTaP doses, there were significant increases in the slope of overall delay (0.15%, *p* = 0.001), a significant and immediate reduction in the level (4.12%, *p* = 0.021) but a significant increase in the slope (0.18%, *p* = 0.001) of delay ≤ 3 months, and a significant and immediate increase in the level of delay > 3 months (4.72%, *p* = 0.012) following the vaccine incident. However, we did not find any significant change in levels and slopes of other NIP vaccination delays after the vaccine incident. For weekly proportions of delayed non-NIP vaccine doses, we found a significant and immediate reduction in the levels of overall delay (5.14%, *p* = 0.019) and delay > 3 months (4.21%, *p* = 0.020) after the vaccine incident, but no significant change was found on the slopes. A closer look at the trend before and after the incident can be found in Supplementary Fig. 2, where we set more cut-off points to display the time lag effect.

Table 2 compares the total proportion of delayed doses by vaccine type before and after the vaccine incident, including short-term and long-term vaccination delays. Following the incident, significant increases in the proportions of overall delayed doses (26.55% to 28.71%, *p* = 0.000), doses delayed ≤ 3 months (19.38% to 20.26%, *p* = 0.012), and doses delayed > 3 months (7.17% to 8.45%, *p* = 0.000) were observed for overall surveyed NIP vaccines. Among them, DTaP vaccines showed the highest increases in vaccination delay after the incident: the proportion of overall delayed DTaP doses increased from 26.29% to 34.33% (*p* = 0.000), the proportion of doses delayed ≤ 3 months increased from 19.12% to 22.51% (*p* = 0.000), and the proportion of doses delayed > 3 months increased from 7.17% to 11.82% (*p* = 0.000). By scaling these vaccination delays nationally, the Changchun Changshen vaccine incident would lead to an extra 2,062,168 doses delayed ≤ 3 months and 2,828,638 doses delayed > 3 months for the four-dose DTaP vaccines when extrapolating these delayed proportions to the 2018 national live birth cohort in China (15,207,729 live newborns)^28^.

**Table 2:** Proportion of delayed doses (%) by vaccine type before and after the vaccine incident.

Vaccine type^a^	Number of doses in schedule^b^		Proportion of overall delayed doses ^c^, %			Proportion of doses delayed ≤ 3 months ^d^, %			Proportion of doses delayed > 3 months ^e^, %		
	Before incident	After incident	Before incident	After incident	*p*-value^f^	Before incident	After incident	*p*-value^f^	Before incident	After incident	*p*-value^f^
NIP	37 602	19 797	26.55	28.71	0.000	19.38	20.26	0.012	7.17	8.45	0.000
DTaP (4-dose)	10 155	5 145	26.29	34.33	0.000	19.12	22.51	0.000	7.17	11.82	0.000
NIP (except DTaP)	27 447	14 652	26.65	26.73	0.856	19.48	19.47	0.989	7.17	7.26	0.739
BCG	3 434	1 719	17.53	23.04	0.000	15.49	21.35	0.000	2.04	1.69	0.386
Hep B (3-dose)	9 411	4 947	18.07	18.25	0.791	14.12	15.12	0.106	3.95	3.13	0.013
Polio (3-dose)	9 223	4 828	20.20	16.11	0.000	16.45	13.73	0.000	3.75	2.38	0.000
MMR	1 491	908	38.10	39.65	0.449	24.35	26.65	0.207	13.75	13.00	0.600
JE	2 397	1 342	64.28	62.37	0.242	39.42	34.28	0.002	24.86	28.09	0.031
Hep A	1 491	908	69.76	70.82	0.581	44.27	41.08	0.126	25.49	29.74	0.023
Non-NIP	11 874	6 306	77.36	76.44	0.161	7.17	7.03	0.723	70.19	69.41	0.277
Hib (4-dose)	10 194	5 388	81.12	79.96	0.081	7.71	7.35	0.419	73.41	72.61	0.284
Varicella	1 680	918	54.52	55.77	0.541	3.87	5.12	0.134	50.65	50.65	1.000

^a^*NIP* National Immunization Program, *DTaP* Diphtheria-tetanus-pertussis, *BCG* Bacillus Calmette-Guerin, *HepB* Hepatitis B, *MMR* Measles-mumps-rubella, *JE* Japanese encephalitis, *HepA* Hepatitis A, *Hib* Haemophilus influenza type b.

^b^Vaccines with multiple doses were analyzed by dose and summed up, so their number of doses in schedule was nearly three- or four-fold as those vaccines with only one dose. A dose was considered as timely administered if it was vaccinated within the vaccination window, no matter how many doses the vaccine includes.

^c^Vaccine doses not administrated in the vaccination window, including doses delayed ≤ 3 months (d) and > 3 months (e).

^d^Vaccine doses delayed but vaccinated within 3 months beyond the vaccination window.

^e^Vaccine doses delayed for more than 3 months beyond the vaccination window, including those finally being vaccinated after 3 months and those have not been vaccinated until the survey time.

^f^Chi-square test comparing the proportions of delayed doses before and after the vaccine incident.

For the other six NIP vaccines in total, no significant change was found in the proportion of overall delayed doses after the incident. When separating different types of other NIP doses, no significant changes were found for vaccination delays of HepB vaccine, MMR, JE, and HepA vaccine, whereas we found a significant increase in the proportion of delayed BCG doses (17.53%–23.04%, *p* = 0.000) but a significant decline for Polio doses (20.20%–16.11%, *p* = 0.000) following the incident. Besides, no significant changes were observed for vaccination delays of two non-NIP vaccines, i.e. Hib and Varicella vaccines. The sensitivity analysis with a restricted study period (Jul 2017–May 2018 and Jul 2018–May 2019) indicated similar results (Supplementary Table 4).

### Choice of different vaccines containing DTaP antigens before and after the vaccine incident

Table 3 shows the before-and-after comparison for the proportions of administered doses out of doses in schedule and proportions of delayed doses out of administered doses for DTaP vaccines in total, and for different vaccines containing DTaP antigens separately. Following the vaccine incident, the proportion of administered doses in total significantly decreased from 98.79% to 94.52%, and among those administered doses, the proportion of delayed doses significantly increased from 25.39% to 30.52%. Especially, the government-funded but incident-involved trivalent DTaP vaccine experienced a greater drop in the proportion of administered doses by 8.28 percentage points (from 87.23% to 78.95%) and a greater increase in the proportion of delayed doses by 6.73 percentage points (from 26.11% to 32.84%). On the contrary, self-paid quadrivalent DTaP-Hib or pentavalent DTaP-IPV/Hib vaccine experienced a significant increase in the proportion of administered doses by 4.01 percentage points (from 11.56% to 15.57%) although no significant change was found for the proportion of delayed doses. When looking into the first to fourth DTaP doses separately, similar results were found for each dose. In additional analyses, we found that factors such as families with only one child, higher guardian’s education level, higher quintiles of per capita monthly income, and urban residence were potential indicators associated with the decision to procure self-paid vaccines (Supplementary Table 5).

**Table 3:** Comparing guardians’ choice of different vaccines containing DTaP antigens before and after the vaccine incident.

Vaccines containing DTaP antigens	Number of doses in schedule		Number of doses administered		Proportion of administered doses out of doses in schedule, %			Number of doses delayed		Proportion of delayed doses out of administered doses, %		
	Before incident	After incident	Before incident	After incident	Before incident	After incident	*p*-value^a^	Before incident	After incident	Before incident	After incident	*p*-value^a^
Overall 4 doses												
Total	10 155	5 145	10 032	4 863	98.79	94.52	0.000	2 547	1 484	25.39	30.52	0.000
Trivalent^b^			8 858	4 062	87.23	78.95	0.000	2 313	1 334	26.11	32.84	0.000
Quadrivalent/Pentavalent^c^			1 174	801	11.56	15.57	0.000	234	150	19.93	18.73	0.506
Dose 1												
Total	3 250	1 636	3 239	1 604	99.66	98.04	0.000	698	480	21.55	29.93	0.000
Trivalent			2 852	1 317	87.75	80.50	0.000	639	441	22.41	33.49	0.000
Quadrivalent/Pentavalent			387	287	11.91	17.54	0.000	59	39	15.25	13.59	0.546
Dose 2												
Total	2 839	1 308	2 826	1 264	99.54	96.64	0.000	360	222	12.74	17.56	0.000
Trivalent			2 485	1 045	87.53	79.89	0.000	332	201	13.36	19.23	0.000
Quadrivalent/Pentavalent			341	219	12.01	16.74	0.000	28	21	8.21	9.59	0.573
Dose 3												
Total	2 577	1 296	2 553	1 218	99.07	93.98	0.000	528	268	20.68	22.00	0.352
Trivalent			2 260	1 031	87.70	79.55	0.000	486	248	21.50	24.05	0.103
Quadrivalent/Pentavalent			293	187	11.37	14.43	0.006	42	20	14.33	10.70	0.246
Dose 4												
Total	1 489	905	1 414	777	94.96	85.86	0.000	961	514	67.96	66.15	0.387
Trivalent			1 261	669	84.69	73.92	0.000	856	444	67.88	66.37	0.499
Quadrivalent/Pentavalent			153	108	10.28	11.93	0.207	105	70	68.63	64.81	0.519

^a^Chi-square test comparing the proportions of administered or delayed doses before and after the vaccine incident.

^b^Trivalent vaccine was involved in the Changchun Changsheng vaccine incident and was funded by National Immunization Programme.

^c^Quadrivalent DTaP-Hib and pentavalent DTaP-IPV/Hib vaccines were not involved in the Changchun Changsheng vaccine incident and were self-paid vaccines outside of National Immunization Programme.

### Association between the vaccine incident and vaccination delay

Controlling for socio-demographic characteristics and clustering standard errors at the individual level, doses scheduled to be administered after the vaccine incident remained a strong predictor of higher odds of delays for both DTaP vaccine (OR: 3.49, 95% CI: 3.08–3.96) and other six NIP vaccines as a whole (OR: 2.76, 95% CI: 2.55–2.99), while no significant association was found between the vaccine incident and non-NIP vaccination (Table 4). Similar results were found when conducting hierarchical logistic regression clustering at the provincial level (Supplementary Table 6). When looking into its associations with vaccination delays for the other six NIP vaccines (excluding DTaP) separately, significantly positive associations were found for BCG and HepB vaccines, but not for Polio, MMR, JE, and HepA vaccines (Supplementary Tables 7, 8).

**Table 4:** Association between the vaccine incident and vaccination delay by multivariate logistic regressions.

Variables	DTaP dose delayed vs not (*n* = 15,300)		NIP dose (except DTaP) delayed vs not (*n* = 42,099)		Non-NIP dose delayed vs not (*n* = 18,180)	
	OR	95% CI	OR	95% CI	OR	95% CI
Scheduled time for each dose						
Before vaccine incident	Ref.		Ref.		Ref.	
After vaccine incident	3.49**	(3.08, 3.96)	2.76**	(2.55, 2.99)	0.92	(0.81, 1.05)
Child’s age (years)						
< 1	Ref.		Ref.		Ref.	
1–2	1.25**	(1.08, 1.44)	2.71**	(2.44, 3.01)	0.78*	(0.65, 0.94)
2–3	3.17**	(2.68, 3.74)	5.60**	(4.96, 6.33)	1.08	(0.88, 1.31)
3–5	3.99**	(3.34, 4.78)	6.49**	(5.72, 7.37)	0.91	(0.74, 1.13)
Child’s gender						
Female	Ref.		Ref.		Ref.	
Male	1.15**	(1.06, 1.25)	1.07*	(1.01, 1.13)	1.22**	(1.10, 1.36)
Only one child in a family	0.72**	(0.66, 0.79)	0.86**	(0.81, 0.92)	0.69**	(0.62, 0.78)
Guardian’s age (years)						
< 30	Ref.		Ref.		Ref.	
30–39	0.94	(0.85, 1.04)	0.99	(0.92, 1.07)	0.84*	(0.73, 0.97)
40–49	0.75**	(0.62, 0.89)	0.94	(0.83, 1.06)	0.59**	(0.47, 0.74)
≥ 50	0.65*	(0.45, 0.92)	0.91	(0.73, 1.12)	0.56**	(0.36, 0.87)
Guardian’s relationship with the child						
Mother	Ref.		Ref.		Ref.	
Father	1.12	(1.00, 1.25)	1.07**	(0.99, 1.16)	1.18*	(1.01, 1.37)
Grandparent	1.20	(0.86, 1.68)	1.02	(0.84, 1.25)	1.18	(0.77, 1.79)
Ethnic groups						
Han	Ref.		Ref.		Ref.	
Minorities	1.28**	(1.08, 1.53)	0.98	(0.86, 1.12)	1.21	(0.95, 1.54)
Guardian’s education level						
Elementary school or below	Ref.		Ref.		Ref.	
Middle school	0.95	(0.81, 1.12)	1.02	(0.91, 1.14)	0.76*	(0.61, 0.95)
Senior high school or technical school	0.95	(0.80, 1.12)	1.00	(0.89, 1.13)	0.87	(0.69, 1.11)
Three-year college or associate degree	0.90	(0.75, 1.09)	0.99	(0.86, 1.13)	0.70**	(0.54, 0.91)
Bachelor’s degree or above	0.77*	(0.64, 0.94)	0.95	(0.82, 1.08)	0.58**	(0.45, 0.75)
Quintiles of per capita monthly income						
Quintile 1 (CNY 0–1000)	Ref.		Ref.		Ref.	
Quintile 2 (CNY 1001–1600)	0.98	(0.86, 1.12)	0.94	(0.85, 1.03)	0.77**	(0.64, 0.92)
Quintile 3 (CNY 1601–2400	0.96	(0.84, 1.10)	0.92**	(0.84, 1.01)	0.69**	(0.58, 0.83)
Quintile 4 (CNY 2401–3750)	0.99	(0.86, 1.14)	0.97	(0.88, 1.07)	0.67**	(0.56, 0.81)
Quintile 5 (CNY > 3751)	0.98	(0.84, 1.15)	0.94	(0.84, 1.04)	0.53**	(0.43, 0.64)
Status of residence						
Local resident	Ref.		Ref.		Ref.	
Inter-city migrant	1.14*	(1.03, 1.26)	1.08*	(1.01, 1.17)	1.29**	(1.11, 1.49)
Place of residence						
Rural	Ref.		Ref.		Ref.	
Urban	0.99	(0.90, 1.09)	1.11**	(1.03, 1.18)	0.60**	(0.53, 0.68)
Province						
Shandong	Ref.		Ref.		Ref.	
Beijing	0.80*	(0.67, 0.97)	0.76**	(0.68, 0.86)	6.55**	(4.55, 9.45)
Chongqing	1.07	(0.89, 1.29)	1.72**	(1.53, 1.93)	0.80	(0.64, 1.00)
Gansu	1.17	(0.96, 1.43)	0.77**	(0.67, 0.89)	2.28**	(1.64, 3.19)
Guangdong	1.15	(0.96, 1.39)	0.96	(0.86, 1.09)	1.39**	(1.10, 1.77)
Henan	0.79*	(0.65, 0.96)	0.81**	(0.71, 0.93)	0.32**	(0.26, 0.40)
Jiangxi	0.79*	(0.65, 0.96)	0.90	(0.79, 1.02)	0.65**	(0.52, 0.82)
Jilin	0.81*	(0.67, 0.98)	0.83**	(0.73, 0.93)	5.37**	(4.10, 7.03)
Yunnan	1.14	(0.94, 1.37)	1.17*	(1.04, 1.32)	0.72**	(0.57, 0.90)
Shanghai	0.38**	(0.31, 0.47)	0.26**	(0.22, 0.31)	0.31**	(0.25, 0.39)

*DTaP* Diphtheria-tetanus-pertussis, *NIP* National Immunization Program, *CNY* Chinese Yuan, 1 CNY = 0.14496 USD in 2019.

In the regressions, standard errors are clustered at the individual child level. *OR* Odds ratio, *CI* Confidence interval. **p < 0.01, *p < 0.05.

We further investigated its associations with short-term and long-term vaccination delays by multinomial logistic regressions, and found that the vaccine incident had a greater influence on long-term delay than short-term delay for both DTaP vaccine and other NIP vaccines (Supplementary Tables 9–11). Specifically, DTaP doses scheduled after the vaccine incident had significantly higher relative risks of delays lasting ≤ 3 months (RRR: 2.64, 95% CI: 2.30–3.04) and > 3 months (RRR: 6.77, 95% CI: 5.54-8.27) than doses scheduled before the vaccine incident, so did NIP doses except for DTaP in terms of delays lasting ≤ 3 months (RRR: 2.31, 95% CI: 2.12–2.52) and > 3 months (RRR: 4.31, 95% CI: 3.77–4.93).

## Discussion

This is the first to assess the impacts of the Changchun Changsheng vaccine incident on real-world childhood vaccination behaviors using vaccination records. There is also little literature examining vaccination behavior using individuals’ vaccination records for various vaccine incidents across the world. Significantly increased proportions of vaccination delay following the vaccine incident were observed for the DTaP vaccine and some other NIP vaccines, while no significant changes were observed for non-NIP vaccines. After the incident, the proportion of administered government-funded trivalent DTaP doses declined, while that of self-paid quadrivalent DTaP-Hib or pentavalent DTaP-IPV/Hib doses increased accordingly.

Among all surveyed vaccines, the incident-involved DTaP vaccines experienced the biggest rise in the proportion of delayed doses (from 26% to 34%) following the vaccine incident, which was mainly driven by long-term vaccination delay. The risk of long-term delay of DTaP vaccination (from 7% to 12%) increased to a larger extent compared to short-term delay (from 19% to 22%). These indicated that the vaccine incident was negatively associated with timely vaccination, especially for the incident-involved DTaP vaccines. Our findings were consistent with research on other vaccine incidents worldwide using aggregated vaccination data. Rumors about the safety of oral polio vaccine led to an eleven-month boycott of polio vaccine and a substantial decrease in its coverage with only 13% of children aged 12–23 months fully vaccinated in Nigeria in 2003–2004, reaching the lowest point since 1998^29,30^. This polio vaccine incident finally led to a resurgence of wild poliovirus transmission in Nigeria and other 20 previously polio-free countries by 2007^31^. In China, after widespread media reports of infant deaths following HepB administration in December 2013, the use of HepB vaccines for newborns in ten provinces decreased by 18.6%, from 53 653 doses the week before the incident to 43 688 doses during the week that HepB from the implicated company was suspended^7^. Therefore, increases in vaccination delay following perceived safety signals that our study found can challenge and disrupt ongoing vaccination programmes.

Our results also demonstrated a shift in guardians’ choice between different vaccines containing DTaP antigens following the vaccine incident. In the Changchun Changsheng vaccine incident, substandard vaccines were only reported for trivalent DTaP vaccines which are provided for free in the NIP, but were not reported for quadrivalent DTaP-Hib or pentavalent DTaP-IPV/Hib vaccines which rely on out-of-pocket payments by the guardians. We found that after the vaccine incident, guardians reduced the use of trivalent DTaP vaccines, but increased the use of quadrivalent DTaP-Hib or pentavalent DTaP-IPV/Hib vaccines accordingly. This was consistent with a previous study which found that seven months after the Changchun Changsheng vaccine incident, 10.80% of parents in Guangzhou city were reported to choose self-paid vaccines in general over government-provided vaccines for their children^32^. If no switch from low-efficacy trivalent DTaP vaccines to quadrivalent DTaP-Hib or pentavalent DTaP-IPV/Hib vaccines, there would be more and/or longer delays of DTaP vaccination. This shift in guardians’ choice due to the vaccine incident revealed a public avoidance of incident-involved vaccines, despite imposing a large financial burden on the families involved. In 2018, the procurement price was 39 US Dollars for a quadrivalent DTaP-Hib dose and 84 US Dollars for a pentavalent DTaP-IPV/Hib dose. This burden may be mitigated by appropriate government action to ensure the safety of government-provided vaccines, the timeliness of vaccination service delivery, and the accurate communication of key information to the public. Moreover, sufficient preparation is also necessary for the demand surges for self-paid alternative vaccines.

Fortunately, following the 2018 Changchun Changsheng vaccine incident, revaccination was immediately scheduled for children who had received substandard DTaP vaccines, and the Chinese government passed the first Vaccine Administration Act, which aims to strengthen government supervision of the whole process from vaccine development, production to distribution^12^. These government responses may partly offset the negative impacts of this incident. Without government remedy and respondents’ switch to self-paid alternative vaccines, we may even find more and/or longer delays of DTaP vaccination.

It is necessary to early prepare and timely respond to vaccine incidents, especially facing public health emergencies. During the COVID-19 pandemic, COVID-19 vaccine-related events frequently occurred, and several vaccines have been suspended or withdrawn, which jeopardized public trust in vaccination and hindered the global efforts to control the pandemic^33^. Immediate actions and adequate responses are required to curb its negative impacts. An effective surveillance system targeting vaccine safety may help early warn potential safety risks during mass vaccination. When vaccine incidents occur, the government should conduct thorough investigations into the incidents and promptly implement measures to revise the vaccination plans. At the same time, transparent internal and external communication is crucial for properly responding to vaccine incidents. Effective and precise communication of existing information by officials has the potential to foster public confidence, irrespective of the ongoing nature of the investigation^34^. Additionally, proactive measures should be taken to counteract the dissemination of misinformation^35^.

This study has several limitations. First, we only conducted the cross-sectional survey after the vaccine incident and no survey data are available before it, although we had access to vaccination records before the incident. Second, around 20% of participants declined to provide their complete vaccination records (19.26%) or the records were illegible due to blurred pictures (1.35%), which may have led to selection bias. However, the sex and age distribution among children included in this study were similar to the Chinese population eligible for vaccination. Third, potential bias may be introduced when conducting vaccination-related surveys in vaccination centers since guardians who decided to reject all vaccines following the vaccine incident would not be part of the sampling frame (they would not appear at the vaccination center), so our findings may underestimate the impact of the incident. However, this bias would be of limited magnitude as the coverage rate of more than one NIP dose is nearly 100% in China in 2019^21^. Fourth, non-vaccination cannot be determined using survey data or vaccination records since young children may finally get vaccinated after the survey time. Therefore, we measured long-term delays of vaccination encompassing individuals who remained unvaccinated until the survey time, instead of focusing solely on non-vaccination, and substantial delays in vaccination may lead to a heavy burden of vaccine-preventable diseases.

## Conclusion

The 2018 Changchun Changsheng vaccine incident in China was associated with an increase in the proportions of delayed vaccine doses, especially for DTaP vaccines that were involved in the incident. By scaling nationally, there would be an estimated extra 2.1 million doses delayed ≤ 3 months and 2.8 million doses delayed >3 months or even unvaccinated for the four-dose DTaP vaccine in the year following this incident. After the vaccine incident, more guardians chose the relatively more expensive private-market substitutes containing DTaP elements over government-funded DTaP vaccines involved in the incident, imposing a financial burden on families. This study evaluated the changes in individuals’ vaccination behavior, which might provide policy implications for potential vaccine incidents in the future, particularly in the era of the COVID-19 pandemic with mass vaccination.

### Supplementary information

Supplementary Information

Reporting Summary

## Data Availability

Z.H., X.L., and H.F. had full access to all the data in the study and take responsibility for the integrity of the data. Partial data are available in supplementary information, including vaccination window (Supplementary Table 1) and aggregated data on vaccine-specific vaccination timeliness (Supplementary Table 2, 4, Supplementary Fig. 2). Individual data cannot be shared openly to protect study participant privacy, and readers can contact HF (corresponding author) for other data on reasonable request.

## Code availability

Z.H., X.L., and H.F. take responsibility for the accuracy of the data analysis. Readers can access data analysis codes from 10.5281/zenodo.8158679 using Stata version 17.0^26^.
