# Supplementary material for: Assessing the impact of the 2018 Changchun Changsheng vaccine incident on childhood vaccination in China
Source: Commun Med (Lond). 2023 Aug 22;3:114. doi: 10.1038/s43856-023-00339-0 (PMC10444794; doi:10.1038/s43856-023-00339-0)
Supplement: Supplementary file 1 — Supplementary Information [file 43856_2023_339_MOESM1_ESM.docx]

**Supplementary Information**

[Supplementary Note 1. Description of the multistage sampling cluster method and study sites](#_Toc669892108)

[Supplementary Table 1. Vaccination window of vaccines immunized before 18 months of age in China](#_Toc1767381582)

[Supplementary Table 2. Proportion of doses delayed >3 months which were finally vaccinated before the survey time or have not been vaccinated until the survey time before and after the vaccine incident](#_Toc388443370)

[Supplementary Table 3. Relative changes in levels and trends of vaccination delay attributable to vaccine incident estimated by interrupted time series analysis](#_Toc217432710)

[Supplementary Figure 2. Weekly proportions of delayed doses by vaccine type before and after vaccine incident with more cut-off points](#_Toc1521873423)

[Supplementary Table 4. Proportion of delayed doses (%) by vaccine type before and after the vaccine incident with equal time periods](#_Toc1596384591)

[Supplementary Table 5. Factors associated with guardians’ choice of quadrivalent DTaP-Hib or pentavalent DTaP-IPV/Hib vaccines in DTaP vaccination by multivariate logistic regressions](#_Toc1922618966)

[Supplementary Table 6. Association between the vaccine incident and vaccination delays by hierarchical logistic regressions clustering at the provincial level](#_Toc270525153)

[Supplementary Table 7. Association between the vaccine incident and vaccination delays for BCG, Hep B, and Polio vaccines by multivariate logistic regressions](#_Toc493365772)

[Supplementary Table 8. Association between the vaccine incident and vaccination delays for MMR, JE, and Hep A vaccines by multivariate logistic regressions](#_Toc564168937)

[Supplementary Table 9. Association between the vaccine incident and DTaP vaccine dose delays by multinomial logistic regression (Base case: DTaP doses not delayed)](#_Toc847022654)

[Supplementary Table 10. Association between the vaccine incident and NIP vaccine dose (except DTaP) delays by multinomial logistic regression (Base case: NIP doses not delayed)](#_Toc240649815)

[Supplementary Table 11. Association between the vaccine incident and non-NIP vaccine dose delays by multinomial logistic regression (Base case: non-NIP doses not delayed)](#_Toc889733404)

# **Supplementary Note 1. Description of the multistage sampling cluster method and study sites**

In this study, children aged 6-59 months were enrolled through a **multistage cluster sampling method**:

**First**, seven provinces (Jilin, Henan, Jiangxi, Shandong, Guangdong, Gansu, and Yunnan) and three provincial-level cities (Beijing, Shanghai, and Chongqing) were selected based on China’s Division of Central and Local Financial Governance and Expenditure Responsibilities in the Healthcare Sector (http://www.gov.cn/zhengce/content/2018-08/13/content_5313489.htm), which stratifies the 31 provinces/provincial-level cities into five layers according to the socio-economic development and abilities of local governments. Moreover, the distance between different provinces and Shandong province (where the event outbroke) was also one of the factors we considered when determining the sample provinces. In terms of geographical location and socioeconomic development, ten provinces/provincial-level cities (3, 3, 1, 1, and 2 in each layer) were chosen to represent different regions of China, with their ranks of 2018 per capita GDP (e.g., 1/31) recorded in the following map figure.

**Second**, a capital city and a non-capital city were selected in each province. For provincial-level cities, an economically developed district and a less-developed district were selected accordingly.

**Third**, two subdistricts/counties were chosen in each city or district, among which one was comparatively more developed and the other was less developed in the city or district.

**Fourth**, in each subdistrict/county, three to four communities and the corresponding vaccination centers (based in community health centers) were sampled to represent low (below median), median and high (above median) social-economic strata.

**Fifth**, in each vaccination center, guardians of all children aged 6-59 months visiting the sampled vaccination centers on a given day during the survey period were invited to participate in the survey.

**The minimum sample size** was calculated under the assumption that the predicted proportion of delayed doses is 50%. With an allowable error of 5%, the sample size was set to 384 in each province and 3 840 as a total across ten provinces. In our survey, oversampling was used to consider the potential response rate and integrity of data collected. Actually, 6 668 children were recruited in the survey. Among them, the guardians of 5 384 (80.74%) children agreed to provide their vaccination records, and the records of 5 294 (79.39%) were legible and complete with snapshots of every page.


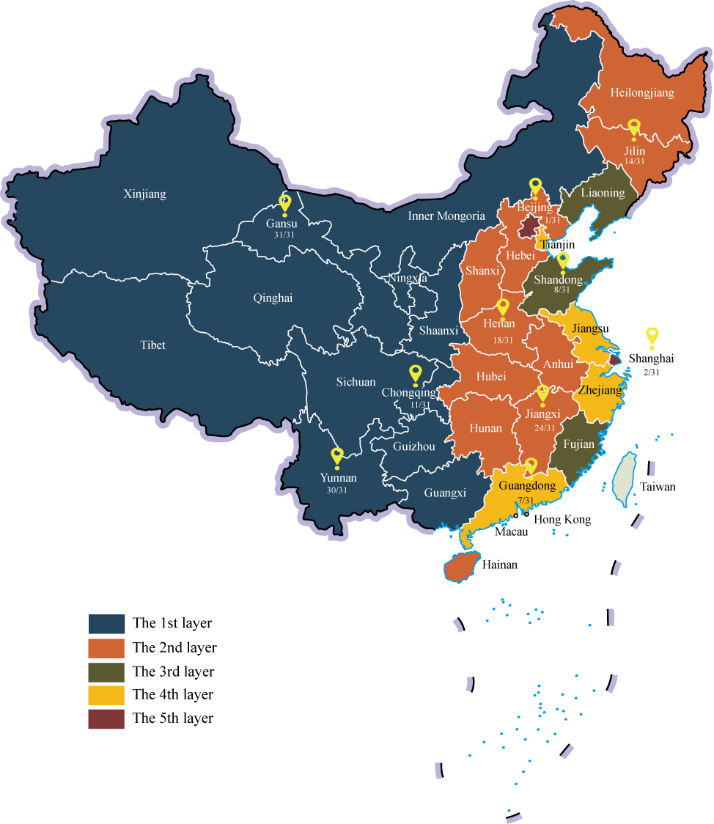


**Supplementary Figure 1. Ten sampled provinces or provincial-level cities in China for the survey.** Ranks of 2018 per capita GDP by province (e.g., 1/31) are marked in the figure.

# **Supplementary Table 1. Vaccination window of vaccines immunized before 18 months of age in China**

| **Vaccine type** | **Vaccination window** | | | | | | | | | | | |
| --- | --- | --- | --- | --- | --- | --- | --- | --- | --- | --- | --- | --- |
|  | **Birth** | **1 m** | **2 m** | **3 m** | **4 m** | **5 m** | **6 m** | **8 m** | **9 m** | **12 m** | **15 m** | **18 m** |
| Diphtheria-tetanus-pertussis (DTaP) vaccine |  |  |  | 1 | 2 | 3 |  |  |  |  |  | 4 |
| Bacillus Calmette-Guerin (BCG) vaccine | 1 |  |  |  |  |  |  |  |  |  |  |  |
| Hepatitis B (HepB) vaccine | 1 | 2 |  |  |  |  | 3 |  |  |  |  |  |
| Polio vaccine |  |  | 1 | 2 | 3 |  |  |  |  |  |  |  |
| Measles-mumps-rubella (MMR) vaccine |  |  |  |  |  |  |  |  |  |  |  | 1 |
| Japanese encephalitis (JE) vaccine |  |  |  |  |  |  |  | 1 |  |  |  |  |
| Hepatitis A (HepA) vaccine |  |  |  |  |  |  |  |  |  |  |  | 1 |
| Haemophilus influenza type b (Hib) conjugate vaccine |  |  |  | 1 | 2 | 3 |  |  |  |  |  | 4 |
| Varicella vaccine |  |  |  |  |  |  |  |  |  | 1 | |  |

# **Supplementary Table 2. Proportion of doses delayed >3 months which were finally vaccinated before the survey time or have not been vaccinated until the survey time before and after the vaccine incident**

| Vaccine type ^a^ | Number of doses in schedule ^b^ | | Proportion of doses delayed >3 months, % | | | Proportion of doses delayed >3 months but finally vaccinated before the survey time, % | | | Proportion of doses delayed >3 months and have not been vaccinated until the survey time, % | | |
| --- | --- | --- | --- | --- | --- | --- | --- | --- | --- | --- | --- |
|  | Before incident | After incident | Before incident | After incident | *p*-value ^c^ | Before incident | After incident | *p*-value ^c^ | Before incident | After incident | *p*-value ^c^ |
| NIP | 37 602 | 19 797 | 7.17 | 8.45 | <0.01 | 6.10 | 5.45 | <0.01 | 1.08 | 3.00 | <0.01 |
| DTaP (4-dose) | 10 155 | 5 145 | 7.17 | 11.82 | <0.01 | 5.96 | 6.34 | 0.36 | 1.21 | 5.48 | <0.01 |
| NIP (except DTaP) | 27 447 | 14 652 | 7.17 | 7.26 | 0.74 | 6.15 | 5.14 | <0.01 | 1.03 | 2.12 | <0.01 |
| BCG | 3 434 | 1 719 | 2.04 | 1.69 | 0.39 | 1.22 | 1.16 | 0.85 | 0.82 | 0.52 | 0.24 |
| Hep B (3-dose) | 9 411 | 4 947 | 3.95 | 3.13 | 0.01 | 3.19 | 2.16 | <0.01 | 0.77 | 0.97 | 0.20 |
| Polio (3-dose) | 9 223 | 4 828 | 3.75 | 2.38 | <0.01 | 3.34 | 1.62 | <0.01 | 0.41 | 0.77 | <0.01 |
| MMR | 1 491 | 908 | 13.75 | 13.00 | 0.60 | 11.27 | 9.03 | 0.08 | 2.48 | 3.96 | 0.04 |
| JE | 2 397 | 1 342 | 24.86 | 28.09 | 0.03 | 23.52 | 22.13 | 0.33 | 1.34 | 5.96 | <0.01 |
| Hep A | 1 491 | 908 | 25.49 | 29.74 | 0.02 | 20.46 | 18.61 | 0.27 | 5.03 | 11.12 | <0.01 |
| Non-NIP | 11 874 | 6 306 | 70.19 | 69.41 | 0.28 | 8.62 | 4.20 | <0.01 | 61.56 | 65.21 | <0.01 |
| Hib (4-dose) | 10 194 | 5 388 | 73.41 | 72.61 | 0.28 | 6.52 | 2.93 | <0.01 | 66.88 | 69.67 | <0.01 |
| Varicella | 1 680 | 918 | 50.65 | 50.65 | 1.00 | 21.37 | 11.66 | <0.01 | 29.29 | 39.00 | <0.01 |
| ^a^ NIP, National Immunization Program; DTaP, Diphtheria-tetanus-pertussis; BCG, Bacillus Calmette-Guerin; HepB, Hepatitis B; MMR, Measles-mumps-rubella, JE, Japanese encephalitis; HepA, Hepatitis A; Hib, Haemophilus influenza type b.  ^b^ Vaccines with multiple doses were analyzed by dose and summed up, so their number of doses in schedule was nearly three- or four-fold as those vaccines with only one dose. A dose was considered as timely administered if it was vaccinated within the vaccination window, no matter how many doses the vaccine includes.  ^c^ Chi-square test comparing the proportions of delayed doses before and after the vaccine incident. | | | | | | | | | | | |

# **Supplementary Table 3. Relative changes in levels and trends of vaccination delay attributable to vaccine incident estimated by interrupted time series analysis**

|  | Weekly proportions of delayed DTaP doses | | | Weekly proportions of delayed NIP vaccine doses (except DTaP) | | | Weekly proportions of delayed non-NIP vaccine doses | | |
| --- | --- | --- | --- | --- | --- | --- | --- | --- | --- |
|  | Overall delay | Delay ≤3 months | Delay >3 months | Overall delay | Delay ≤3 months | Delay >3 months | Overall delay | Delay ≤3 months | Delay >3 months |
| Baseline level | .2706 (.0130)** | .1906 (.0113)** | .0800(.0067)** | .2857 (.0088)** | .2105 (.0073)** | .0751 (.0048)** | .7533 (.0106)** | .0747(.0054)** | .6786 (.0107)** |
| Baseline trend | .0001(.0002) | .0001(.0002) | -.00002 (.0001) | -.0002(.0001) | -.0002(.0001) | .00003(.0001) | .0003(.0002) | -.0001(.0001) | .0004(.0002)* |
| Level change | .0060 (.0238) | -.0412(.0206)* | .0472(.0122)** | .0032(.0161) | -.0027 (.0133) | .0059(.0087) | -.0514 (.0194)** | -.0094(.0099) | -.0421 (.0195)* |
| Trend change | .0015 (.0008)* | .0018 (.0007)** | -.0003 (.0004) | -.00007 (.0005) | .0004 (.0004) | -.0004 (.0003) | .0006(.0006) | .0004 (.0003) | .0002 (.0006) |
| Spring festival | .0738(.0199)** | .0658(.0173)** | .0080(.0102) | .0447(.0135)** | .0476(.0111)** | -.0029(.0073) | .0417(.0162)* | .0270(.0083)** | .0148(.0164) |

A segmented generalized linear model is used, and coefficients and standard errors are presented. “Level change” indicates the change in the level of the outcome immediately after vaccine incident, and “Trend change” indicates the change in the slope of the outcome after vaccine incident. ** p<0.01, * p=<0.05.

# **Supplementary Figure 2. Weekly proportions of delayed doses by vaccine type before and after vaccine incident with more cut-off points**

Data from 104 weeks before the vaccine incident to 44 weeks after the incident, with three lines in each figure representing DTaP doses (blue line), NIP doses excluding DTaP (green line), and non-NIP doses (orange line), respectively. Broken lines represent the observed proportions of delayed doses by week, and straight lines were fitted by a segmented generalized linear model (GLM) with pre-incident and post-incident trends. Vertical broken lines in gray indicated different cut-off points: (1) Nov 4, 2017, 36 weeks prior to the incident, when the first concerns about the vaccines started; (2) Jul 15, 2018, occurrence of the incident; (3) Aug 12, 2018, 4 weeks after the incident; (4) Oct 14, 2018, 13 weeks after the incident.


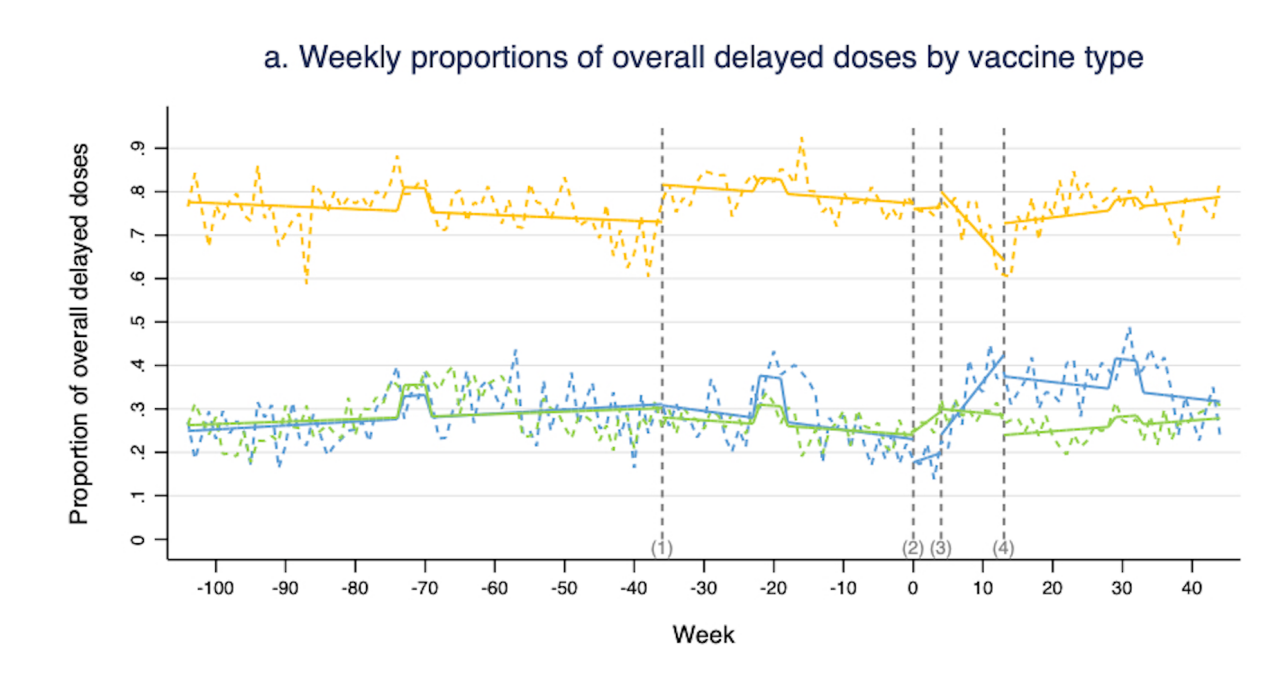


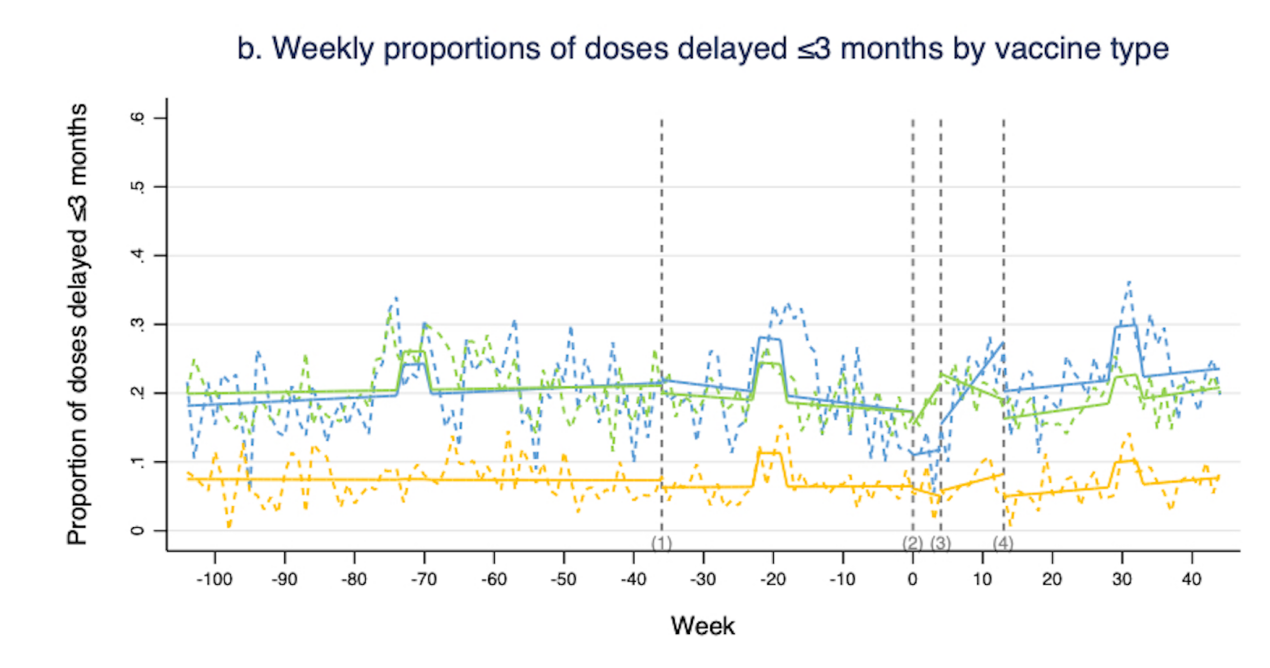


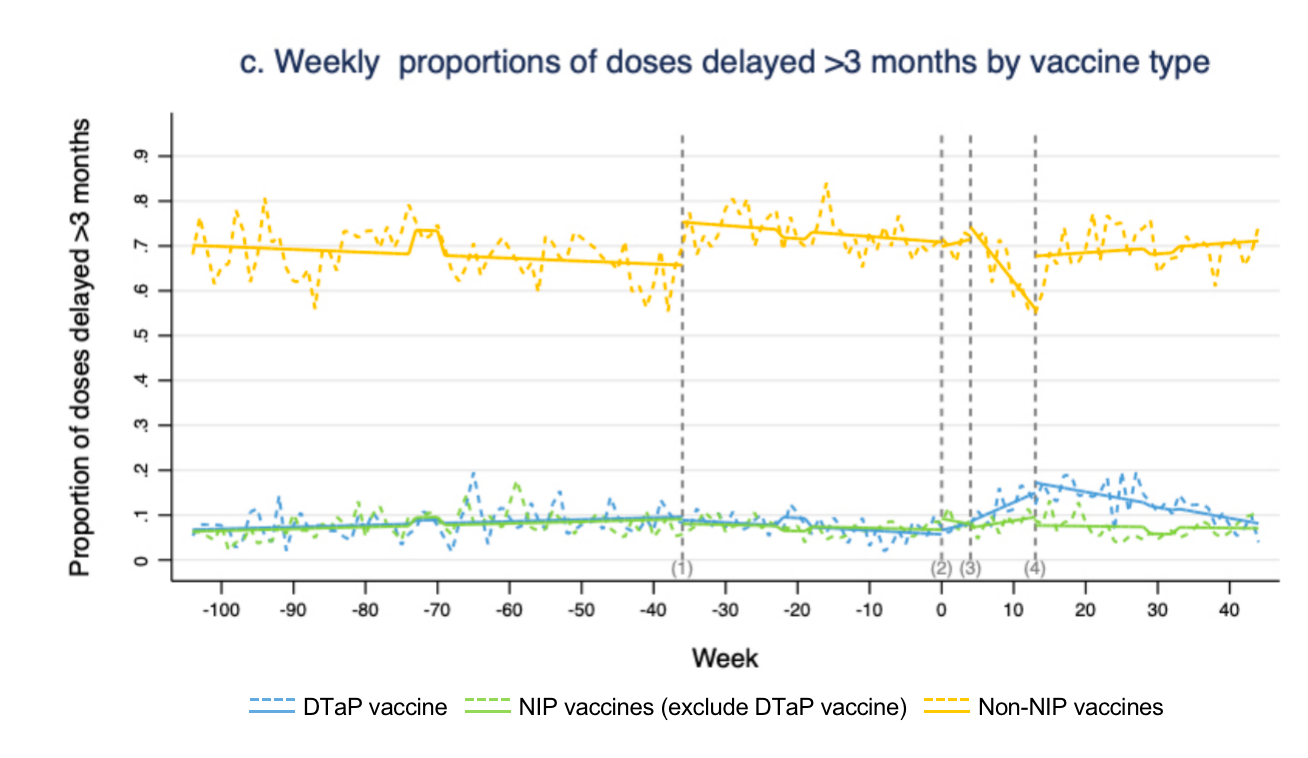


# **Supplementary Table 4. Proportion of delayed doses (%) by vaccine type before and after the vaccine incident with equal time periods**

| Vaccine type ^a^ | Number of doses in schedule ^b^ | | Proportion of overall delayed doses ^c^, % | | | Proportion of doses delayed ≤3 months ^d^, % | | | Proportion of doses delayed >3 months ^e^, % | | |
| --- | --- | --- | --- | --- | --- | --- | --- | --- | --- | --- | --- |
|  | Before incident | After incident | Before incident | After incident | *p*-value ^f^ | Before incident | After incident | *p*-value ^f^ | Before incident | After incident | *p*-value ^f^ |
| NIP | 16059 | 19797 | 27.33 | 28.71 | <0.01 | 19.86 | 20.26 | 0.34 | 7.47 | 8.45 | <0.01 |
| DTaP (4-dose) | 4165 | 5145 | 28.39 | 34.33 | <0.01 | 20.49 | 22.51 | 0.02 | 7.9 | 11.82 | <0.01 |
| NIP (except DTaP) | 11894 | 14652 | 26.96 | 26.73 | 0.68 | 19.64 | 19.47 | 0.73 | 7.32 | 7.26 | 0.85 |
| BCG | 1483 | 1719 | 17.33 | 23.04 | <0.01 | 15.44 | 21.35 | <0.01 | 1.89 | 1.69 | 0.67 |
| Hep B (3-dose) | 4012 | 4947 | 18.37 | 18.25 | 0.89 | 14.28 | 15.12 | 0.27 | 4.09 | 3.13 | 0.02 |
| Polio (3-dose) | 3868 | 4828 | 20.19 | 16.11 | <0.01 | 16.47 | 13.73 | <0.01 | 3.72 | 2.38 | <0.01 |
| MMR | 797 | 908 | 35.38 | 39.65 | 0.07 | 23.71 | 26.65 | 0.16 | 11.67 | 13 | 0.41 |
| JE | 937 | 1342 | 66.06 | 62.37 | 0.07 | 38.31 | 34.28 | 0.04 | 27.75 | 28.09 | 0.86 |
| Hep A | 797 | 908 | 66.63 | 70.82 | 0.06 | 43.79 | 41.08 | 0.26 | 22.84 | 29.74 | <0.01 |
| Non-NIP | 4981 | 6306 | 77.58 | 76.44 | 0.16 | 7.04 | 7.03 | 0.99 | 70.54 | 69.41 | 0.2 |
| Hib (4-dose) | 4249 | 5388 | 81.39 | 79.96 | 0.08 | 7.67 | 7.35 | 0.55 | 73.72 | 72.61 | 0.23 |
| Varicella | 732 | 918 | 55.41 | 55.77 | 0.87 | 3.36 | 5.12 | 0.08 | 52.05 | 50.65 | 0.57 |
| The study period was restricted to Jul 2017-May 2018 (before the incident) and Jul 2018-May 2019 (after the incident). ^a^ NIP, National Immunization Program; DTaP, Diphtheria-tetanus-pertussis; BCG, Bacillus Calmette-Guerin; HepB, Hepatitis B; MMR, Measles-mumps-rubella, JE, Japanese encephalitis; HepA, Hepatitis A; Hib, Haemophilus influenza type b. ^b^ Vaccines with multiple doses were analyzed by dose and summed up, so their number of doses in schedule was nearly three- or four-fold as those vaccines with only one dose. A dose was considered as timely administered if it was vaccinated within the vaccination window, no matter how many doses the vaccine includes. ^c^ Vaccine doses not administrated in the vaccination window, including doses delayed ≤ 3 months (d) and > 3 months (e). ^d^ Vaccine doses delayed but vaccinated within 3 months beyond the vaccination window. ^e^ Vaccine doses delayed for more than 3 months beyond the vaccination window, including those finally being vaccinated after 3 months and those have not been vaccinated until the survey time. ^f^ Chi-square test comparing the proportions of delayed doses before and after the vaccine incident. | | | | | | | | | | | |
|  |  |  |  |  |  |  |  |  |  |  |  |
|  |  |  |  |  |  |  |  |  |  |  |  |
|  |  |  |  |  |  |  |  |  |  |  |  |
|  |  |  |  |  |  |  |  |  |  |  |  |

# **Supplementary Table 5. Factors associated with guardians’ choice of quadrivalent DTaP-Hib or pentavalent DTaP-IPV/Hib vaccines in DTaP vaccination by multivariate logistic regressions**

| Variables | Overall | | Doses scheduled before vaccine incident | | Doses scheduled after vaccine incident | |
| --- | --- | --- | --- | --- | --- | --- |
|  | OR | 95% CI | OR | 95% CI | OR | 95% CI |
| Scheduled time for each dose |  |  |  |  |  |  |
| Before vaccine incident | Ref. |  | - | - | - | - |
| After vaccine incident | 1.26 * | (1.03, 1.54) | - | - | - | - |
| Child’s age (years) |  |  |  |  |  |  |
| <1 | Ref. |  | Ref. |  | Ref. |  |
| 1-2 | 0.74 * | (0.56, 0.98) | 2.73 | (0.52, 14.40) | 0.71 * | (0.53, 0.96) |
| 2-3 | 0.56 ** | (0.40, 0.78) | 1.97 | (0.37, 10.43) | 0.55 ** | (0.39, 0.77) |
| 3-5 | 0.51 ** | (0.37, 0.73) | 1.78 | (0.34, 9.36) | 2.42 | (0.53, 10.99) |
| Child’s gender |  |  |  |  |  |  |
| Female | Ref. |  | Ref. |  | Ref. |  |
| Male | 0.88 | (0.72, 1.07) | 0.83 | (0.64, 1.07) | 0.96 | (0.74, 1.25) |
| Only one child in a family | 1.54 ** | (1.24, 1.90) | 1.51 ** | (1.14, 1.99) | 1.55 ** | (1.17, 2.05) |
| Guardian’s age (years) |  |  |  |  |  |  |
| <30 | Ref. |  | Ref. |  | Ref. |  |
| 30-39 | 1.21 | (0.94, 1.56) | 1.41 | (0.98, 2.03) | 1.02 | (0.74, 1.41) |
| 40-49 | 2.48 ** | (1.62, 3.80) | 3.08 ** | (1.77, 5.34) | 1.90 * | (1.07, 3.39) |
| ≥50 | 2.56 * | (1.09, 6.04) | 4.02 * | (1.17, 13.84) | 1.82 | (0.60, 5.50) |
| Guardian’s relationship with the child |  |  |  |  |  |  |
| Mather | Ref. |  | Ref. |  | Ref. |  |
| Father | 0.72 * | (0.55, 0.95) | 0.59 ** | (0.41, 0.85) | 0.96 | (0.68, 1.37) |
| Grandparent | 0.98 | (0.42, 2.28) | 0.69 | (0.20, 2.40) | 1.36 | (0.48, 3.81) |
| Ethnic groups |  |  |  |  |  |  |
| Han | Ref. |  | Ref. |  | Ref. |  |
| Minorities | 0.48 ** | (0.27, 0.83) | 0.44 * | (0.22, 0.90) | 0.55 | (0.27, 1.13) |
| Guardian’s education level |  |  |  |  |  |  |
| Elementary school or below | Ref. |  | Ref. |  | Ref. |  |
| Middle school | 1.34 | (0.76, 2.35) | 1.15 | (0.57, 2.31) | 1.84 | (0.83, 4.05) |
| Senior high school or technical school | 2.15 ** | (1.20, 3.84) | 2.07 * | (1.01, 4.25) | 2.67 * | (1.20, 5.95) |
| Three-year college or associate degree | 3.31 ** | (1.82, 6.03) | 3.45 ** | (1.64, 7.27) | 3.40 ** | (1.49, 7.77) |
| Bachelor’s degree or above | 4.88 ** | (2.68, 8.86) | 4.65 ** | (2.17, 9.95) | 6.12 ** | (2.73, 13.69) |
| Quintiles of per capita monthly income |  |  |  |  |  |  |
| Quintile 1 (CNY 0-1,000) | Ref. |  | Ref. |  | Ref. |  |
| Quintile 2 (CNY 1,001-1,600) | 2.16 ** | (1.36, 3.43) | 2.66 ** | (1.45, 4.88) | 1.50 | (0.82, 2.76) |
| Quintile 3 (CNY 1,601-2,400) | 2.69 ** | (1.75, 4.14) | 2.50 ** | (1.36, 4.59) | 2.85 ** | (1.66, 4.90) |
| Quintile 4 (CNY 2,401-3,750) | 2.99 ** | (1.94, 4.62) | 3.03 ** | (1.66, 5.55) | 2.90 ** | (1.71, 4.91) |
| Quintile 5 (CNY >3,751) | 4.49 ** | (2.88, 6.99) | 3.85 ** | (2.06, 7.18) | 5.81 ** | (3.41, 9.90) |
| Status of residence |  |  |  |  |  |  |
| Local resident | Ref. |  | Ref. |  | Ref. |  |
| Inter-city migrant | 0.88 | (0.68, 1.13) | 0.89 | (0.64, 1.24) | 0.83 | (0.60, 1.15) |
| Place of residence |  |  |  |  |  |  |
| Rural | Ref. |  | Ref. |  | Ref. |  |
| Urban | 4.63 ** | (3.55, 6.02) | 5.75 ** | (3.89, 8.50) | 3.53 ** | (2.53, 4.93) |
| Province |  |  |  |  |  |  |
| Shandong | Ref. |  | Ref. |  | Ref. |  |
| Beijing | 0.42 ** | (0.25, 0.70) | 0.55 | (0.27, 1.14) | 0.29 ** | (0.15, 0.53) |
| Chongqing | 1.58 * | (1.02, 2.45) | 2.21 * | (1.21, 4.06) | 1.07 | (0.62, 1.87) |
| Gansu | 1.99 ** | (1.21, 3.28) | 3.05 ** | (1.55, 5.99) | 1.25 | (0.68, 2.30) |
| Guangdong | 1.55 | (0.99, 2.43) | 2.43 ** | (1.32, 4.47) | 0.91 | (0.51, 1.61) |
| Henan | 3.74 ** | (2.45, 5.72) | 5.27 ** | (2.96, 9.38) | 2.57 ** | (1.44, 4.56) |
| Jiangxi | 1.74 * | (1.13, 2.69) | 1.94 * | (1.03, 3.65) | 1.55 | (0.88, 2.70) |
| Jilin | 0.07 ** | (0.02, 0.19) | 0.08 ** | (0.02, 0.36) | 0.05 ** | (0.01, 0.18) |
| Yunnan | 2.43 ** | (1.51, 3.92) | 4.16 ** | (2.27, 7.62) | 1.07 | (0.55, 2.07) |
| Shanghai | 6.31 ** | (4.19, 9.52) | 8.88 ** | (5.03, 15.67) | 4.54 ** | (2.65, 7.79) |
| NIP, National Immunization Program; CNY, Chinese Yuan, 1 CNY=0.14496 USD in 2019.  In the regressions, standard errors are clustered at the individual child level. OR, odds ratio; CI, confidence interval. ** p<0.01, * p<0.05. | | | | | | |

# **Supplementary Table 6. Association between the vaccine incident and vaccination delays by hierarchical logistic regressions clustering at the provincial level**

| Variables | DTaP dose delayed vs not | | NIP dose (except DTaP) delayed vs not | | Non-NIP dose delayed vs not | |
| --- | --- | --- | --- | --- | --- | --- |
|  | OR | 95% CI | OR | 95% CI | OR | 95% CI |
| Scheduled time for each dose |  |  |  |  |  |  |
| Before vaccine incident | Ref. |  | Ref. |  | Ref. |  |
| After vaccine incident | 3.49 ** | (3.11, 3.91) | 2.76 ** | (2.58, 2.96) | 0.92 | (0.82, 1.03) |
| Child’s age (years) |  |  |  |  |  |  |
| <1 | Ref. |  | Ref. |  | Ref. |  |
| 1-2 | 1.25 ** | (1.10, 1.42) | 2.71 ** | (2.50, 2.94) | 0.79 ** | (0.69, 0.90) |
| 2-3 | 3.16 ** | (2.73, 3.67) | 5.60 ** | (5.09, 6.15) | 1.08 | (0.92, 1.26) |
| 3-5 | 3.98 ** | (3.39, 4.68) | 6.49 ** | (5.87, 7.17) | 0.91 | (0.77, 1.08) |
| Child’s gender |  |  |  |  |  |  |
| Female | Ref. |  | Ref. |  | Ref. |  |
| Male | 1.15 ** | (1.07, 1.24) | 1.07 ** | (1.02, 1.12) | 1.22 ** | (1.13, 1.32) |
| Only one child in a family | 0.72 ** | (0.66, 0.78) | 0.86 ** | (0.82, 0.90) | 0.70 ** | (0.64, 0.76) |
| Guardian’s age (years) |  |  |  |  |  |  |
| <30 | Ref. |  | Ref. |  | Ref. |  |
| 30-39 | 0.94 | (0.86, 1.03) | 0.99 | (0.94, 1.05) | 0.84 ** | (0.76, 0.93) |
| 40-49 | 0.75 ** | (0.64, 0.87) | 0.94 | (0.85, 1.03) | 0.59 ** | (0.51, 0.70) |
| ≥50 | 0.65 ** | (0.48, 0.87) | 0.90 | (0.75, 1.09) | 0.56 ** | (0.41, 0.78) |
| Guardian’s relationship with the child |  |  |  |  |  |  |
| Mother | Ref. |  | Ref. |  | Ref. |  |
| Father | 1.12 * | (1.01, 1.23) | 1.07 * | (1.01, 1.14) | 1.18 ** | (1.06, 1.31) |
| Grandparent | 1.20 | (0.90, 1.59) | 1.02 | (0.86, 1.22) | 1.18 | (0.87, 1.60) |
| Ethnic groups |  |  |  |  |  |  |
| Han | Ref. |  | Ref. |  | Ref. |  |
| Minorities | 1.29 ** | (1.10, 1.51) | 0.98 | (0.89, 1.08) | 1.21 * | (1.01, 1.45) |
| Guardian’s education level |  |  |  |  |  |  |
| Elementary school or below | Ref. |  | Ref. |  | Ref. |  |
| Middle school | 0.95 | (0.82, 1.10) | 1.02 | (0.93, 1.11) | 0.76 ** | (0.65, 0.90) |
| Senior high school or technical school | 0.94 | (0.81, 1.10) | 1.00 | (0.91, 1.10) | 0.87 | (0.73, 1.04) |
| Three-year college or associate degree | 0.90 | (0.76, 1.07) | 0.99 | (0.89, 1.09) | 0.70 ** | (0.58, 0.85) |
| Bachelor’s degree or above | 0.77 ** | (0.65, 0.92) | 0.94 | (0.85, 1.05) | 0.58 ** | (0.48, 0.70) |
| Quintiles of per capita monthly income |  |  |  |  |  |  |
| Quintile 1 (CNY 0-1,000) | Ref. |  | Ref. |  | Ref. |  |
| Quintile 2 (CNY 1,001-1,600) | 0.98 | (0.87, 1.10) | 0.94 | (0.87, 1.01) | 0.77 ** | (0.67, 0.88) |
| Quintile 3 (CNY 1,601-2,400) | 0.96 | (0.85, 1.08) | 0.92 * | (0.86, 0.99) | 0.69 ** | (0.60, 0.79) |
| Quintile 4 (CNY 2,401-3,750) | 0.99 | (0.87, 1.12) | 0.97 | (0.90, 1.04) | 0.67 ** | (0.58, 0.77) |
| Quintile 5 (CNY >3,751) | 0.97 | (0.84, 1.11) | 0.93 | (0.86, 1.02) | 0.53 ** | (0.46, 0.61) |
| Status of residence |  |  |  |  |  |  |
| Local resident | Ref. |  | Ref. |  | Ref. |  |
| Inter-city migrant | 1.13 * | (1.03, 1.24) | 1.08 ** | (1.02, 1.14) | 1.29 ** | (1.16, 1.42) |
| Place of residence |  |  |  |  |  |  |
| Rural | Ref. |  | Ref. |  | Ref. |  |
| Urban | 0.99 | (0.92, 1.08) | 1.11 ** | (1.05, 1.16) | 0.60 ** | (0.55, 0.66) |
| DTaP, Diphtheria-tetanus-pertussis; NIP, National Immunization Program; CNY, Chinese Yuan, 1 CNY=0.14496 USD in 2019. | | | | | | |
| In the regressions, xtmelogit was used to account for clustering at the provincial level. OR, odds ratio; CI, confidence interval. ** p<0.01, * p<0.05. | | | | | | |

#

# **Supplementary Table 7. Association between the vaccine incident and vaccination delays for BCG, Hep B, and Polio vaccines by multivariate logistic regressions**

| Variables | NIP vaccine doses (except DTaP) delayed vs not, OR (95% CI) | | |
| --- | --- | --- | --- |
|  | BCG | Hep B | Polio |
| Scheduled time for each dose |  |  |  |
| Before vaccine incident | Ref. |  |  |
| After vaccine incident | 1.65 ** (1.16, 2.34) | 2.25 ** (1.93, 2.63) | 1.09 (0.92, 1.28) |
| Child’s age (years) |  |  |  |
| <1 | Ref. |  |  |
| 1-2 | 1.08 (0.78, 1.50) | 2.41 ** (2.07, 2.80) | 1.17 (0.99, 1.39) |
| 2-3 | 1.03 (0.67, 1.58) | 3.91 ** (3.16, 4.82) | 1.73 ** (1.39, 2.16) |
| 3-5 | 1.25 (0.82, 1.89) | 3.40 ** (2.76, 4.19) | 1.56 ** (1.26, 1.94) |
| Child’s gender |  |  |  |
| Female | Ref. |  |  |
| Male | 1.07 (0.90, 1.27) | 1.10 * (1.00, 1.20) | 1.14 ** (1.04, 1.26) |
| Only one child in a family | 1.29 * (1.06, 1.56) | 0.86 ** (0.77, 0.95) | 0.78 ** (0.70, 0.87) |
| Guardian’s age (years) |  |  |  |
| <30 | Ref. |  |  |
| 30-39 | 0.93 (0.76, 1.15) | 0.97 (0.86, 1.08) | 1.00 (0.89, 1.13) |
| 40-49 | 1.46 (0.99, 2.14) | 1.01 (0.83, 1.24) | 0.73 ** (0.59, 0.91) |
| ≥50 | 1.33 (0.69, 2.55) | 1.14 (0.79, 1.65) | 0.64 * (0.44, 0.92) |
| Guardian’s relationship with the child |  |  |  |
| Mother | Ref. |  |  |
| Father | 0.96 (0.75, 1.22) | 1.09 (0.96, 1.23) | 1.07 (0.93, 1.22) |
| Grandparent | 0.67 (0.36, 1.25) | 0.84 (0.59, 1.19) | 1.32 (0.94, 1.87) |
| Ethnic groups |  |  |  |
| Han | Ref. |  |  |
| Minorities | 0.99 (0.67, 1.46) | 0.92 (0.74, 1.13) | 1.05 (0.85, 1.28) |
| Guardian’s education level |  |  |  |
| Elementary school or below | Ref. |  |  |
| Middle school | 1.13 (0.80, 1.60) | 1.04 (0.87, 1.25) | 0.91 (0.75, 1.09) |
| Senior high school or technical school | 0.90 (0.62, 1.30) | 1.05 (0.86, 1.27) | 0.89 (0.73, 1.09) |
| Three-year college or associate degree | 1.06 (0.70, 1.59) | 1.04 (0.84, 1.29) | 0.89 (0.72, 1.11) |
| Bachelor’s degree or above | 0.88 (0.58, 1.34) | 1.00 (0.80, 1.24) | 0.84 (0.67, 1.06) |
| Quintiles of per capita monthly income |  |  |  |
| Quintile 1 (CNY 0-1,000) | Ref. |  |  |
| Quintile 2 (CNY 1,001-1,600) | 0.97 (0.74, 1.27) | 0.88 (0.76, 1.01) | 0.94 (0.82, 1.09) |
| Quintile 3 (CNY 1,601-2,400) | 0.96 (0.73, 1.27) | 0.89 (0.77, 1.03) | 0.94 (0.80, 1.10) |
| Quintile 4 (CNY 2,401-3,750) | 1.02 (0.76, 1.36) | 0.94 (0.80, 1.09) | 0.91 (0.77, 1.07) |
| Quintile 5 (CNY >3,751) | 0.82 (0.58, 1.15) | 0.87 (0.73, 1.03) | 0.95 (0.79, 1.14) |
| Status of residence |  |  |  |
| Local resident | Ref. |  |  |
| Inter-city migrant | 1.14 (0.91, 1.44) | 1.08 (0.97, 1.21) | 1.13 (1.00, 1.27) |
| Place of residence |  |  |  |
| Rural | Ref. |  |  |
| Urban | 1.05 (0.87, 1.28) | 1.06 (0.96, 1.18) | 0.95 (0.86, 1.07) |
| NIP, National Immunization Program; DTaP, Diphtheria-tetanus-pertussis; BCG, Bacillus Calmette-Guerin; HepB, Hepatitis B; MMR, Measles-mumps-rubella, JE, Japanese encephalitis; HepA, Hepatitis A; CNY, Chinese Yuan, 1 CNY=0.14496 USD in 2019.  In the regressions, the province variable is controlled, and standard errors are clustered at the individual child level. OR, odds ratio; CI, confidence interval. ** p<0.01, * p<0.05. | | | |

# **Supplementary Table 8. Association between the vaccine incident and vaccination delays for MMR, JE, and Hep A vaccines by multivariate logistic regressions**

| Variables | NIP vaccine doses (except DTaP) delayed vs not, OR (95% CI) | | |
| --- | --- | --- | --- |
|  | MMR | JE | Hep A |
| Scheduled time for each dose |  |  |  |
| Before vaccine incident |  |  |  |
| After vaccine incident | 1.15 (0.82, 1.62) | 0.93 (0.62, 1.38) | 1.32 (0.90, 1.95) |
| Child’s age (years) |  |  |  |
| <1 |  |  |  |
| 1-2 | 0.89 (0.18, 4.32) | 0.70 (0.32, 1.56) | 0.00 ** (0.00, 0.00) |
| 2-3 | 0.76 (0.16, 3.57) | 0.81 (0.35, 1.85) | 0.00 ** (0.00, 0.00) |
| 3-5 | 0.83 (0.17, 3.94) | 0.84 (0.37, 1.92) | 0.00 ** (0.00, 0.00) |
| Child’s gender |  |  |  |
| Female |  |  |  |
| Male | 1.06 (0.89, 1.26) | 1.15 (0.99, 1.33) | 0.92 (0.75, 1.12) |
| Only one child in a family | 0.71 ** (0.58, 0.86) | 0.83 * (0.70, 0.99) | 0.89 (0.72, 1.11) |
| Guardian’s age (years) |  |  |  |
| <30 |  |  |  |
| 30-39 | 0.93 (0.74, 1.16) | 1.01 (0.83, 1.22) | 0.96 (0.74, 1.26) |
| 40-49 | 0.91 (0.64, 1.29) | 0.81 (0.59, 1.12) | 1.00 (0.64, 1.57) |
| ≥50 | 0.80 (0.40, 1.61) | 0.76 (0.42, 1.36) | 0.76 (0.34, 1.71) |
| Guardian’s relationship with the child |  |  |  |
| Mother |  |  |  |
| Father | 1.16 (0.92, 1.46) | 1.09 (0.88, 1.35) | 1.22 (0.93, 1.62) |
| Grandparent | 1.08 (0.55, 2.10) | 1.15 (0.66, 2.00) | 0.92 (0.43, 2.00) |
| Ethnic groups |  |  |  |
| Han |  |  |  |
| Minorities | 1.09 (0.74, 1.59) | 1.07 (0.76, 1.52) | 0.88 (0.57, 1.38) |
| Guardian’s education level |  |  |  |
| Elementary school or below |  |  |  |
| Middle school | 1.02 (0.74, 1.41) | 0.97 (0.73, 1.29) | 1.20 (0.83, 1.74) |
| Senior high school or technical school | 1.07 (0.75, 1.53) | 1.04 (0.76, 1.42) | 1.18 (0.79, 1.76) |
| Three-year college or associate degree | 1.04 (0.70, 1.54) | 0.92 (0.65, 1.29) | 1.17 (0.75, 1.82) |
| Bachelor’s degree or above | 0.88 (0.59, 1.32) | 0.83 (0.59, 1.18) | 1.33 (0.83, 2.12) |
| Quintiles of per capita monthly income |  |  |  |
| Quintile 1 (CNY 0-1,000) |  |  |  |
| Quintile 2 (CNY 1,001-1,600) | 1.12 (0.86, 1.48) | 0.79 * (0.62, 1.00) | 0.95 (0.69, 1.30) |
| Quintile 3 (CNY 1,601-2,400) | 0.91 (0.69, 1.21) | 0.83 (0.65, 1.06) | 0.72 * (0.52, 0.99) |
| Quintile 4 (CNY 2,401-3,750) | 1.15 (0.85, 1.56) | 1.01 (0.78, 1.32) | 0.93 (0.66, 1.31) |
| Quintile 5 (CNY >3,751) | 1.03 (0.74, 1.45) | 1.00 (0.75, 1.34) | 0.86 (0.58, 1.27) |
| Status of residence |  |  |  |
| Local resident |  |  |  |
| Inter-city migrant | 1.20 (0.96, 1.50) | 1.09 (0.89, 1.34) | 1.17 (0.89, 1.55) |
| Place of residence |  |  |  |
| Rural |  |  |  |
| Urban | 1.13 (0.92, 1.38) | 1.59 ** (1.34, 1.88) | 2.37 ** (1.88, 2.99) |
| NIP, National Immunization Program; DTaP, Diphtheria-tetanus-pertussis; BCG, Bacillus Calmette-Guerin; HepB, Hepatitis B; MMR, Measles-mumps-rubella, JE, Japanese encephalitis; HepA, Hepatitis A; CNY, Chinese Yuan, 1 CNY=0.14496 USD in 2019.  In the regressions, the province variable is controlled, and standard errors are clustered at the individual child level. OR, odds ratio; CI, confidence interval. ** p<0.01, * p<0.05. | | | |

# **Supplementary Table 9. Association between the vaccine incident and DTaP vaccine dose delays by multinomial logistic regression (Base case: DTaP doses not delayed)**

| Variables | DTaP doses delayed ≤3 months | | DTaP doses delayed >3 months | | |
| --- | --- | --- | --- | --- | --- |
|  | RRR | 95% CI | RRR | 95% CI | |
| Scheduled time for each dose |  |  |  |  | |
| Before vaccine incident | Ref. |  | Ref. |  | |
| After vaccine incident | 2.64 ** | (2.30, 3.04) | 6.77 ** | (5.54, 8.27) | |
| Child’s age (years) |  |  |  |  | |
| <1 | Ref. |  | Ref. |  | |
| 1-2 | 1.10 | (0.94, 1.28) | 1.62 ** | (1.23, 2.14) | |
| 2-3 | 2.36 ** | (1.98, 2.82) | 6.18 ** | (4.60, 8.29) | |
| 3-5 | 2.91 ** | (2.40, 3.53) | 8.65 ** | (6.21, 12.04) | |
| Child’s gender |  |  |  |  | |
| Female | Ref. |  | Ref. |  | |
| Male | 1.10 * | (1.01, 1.20) | 1.28 ** | (1.11, 1.48) | |
| Only one child in a family | 0.77 ** | (0.70, 0.84) | 0.61 ** | (0.52, 0.72) | |
| Guardian’s age (years) |  |  |  |  | |
| <30 | Ref. |  | Ref. |  | |
| 30-39 | 0.93 | (0.83, 1.03) | 0.98 | (0.82, 1.17) | |
| 40-49 | 0.75 ** | (0.62, 0.91) | 0.73 * | (0.54, 1.00) | |
| ≥50 | 0.64 * | (0.44, 0.93) | 0.67 | (0.38, 1.18) | |
| Guardian’s relationship with the child |  |  |  |  | |
| Mather | Ref. |  | Ref. |  | |
| Father | 1.11 | (0.99, 1.25) | 1.13 | (0.92, 1.38) | |
| Grandparent | 1.36 | (0.95, 1.96) | 0.84 | (0.49, 1.45) | |
| Ethnic groups |  |  |  |  | |
| Han | Ref. |  | Ref. |  | |
| Minorities | 1.25 * | (1.04, 1.50) | 1.36 * | (1.02, 1.81) | |
| Guardian’s education level |  |  |  |  | |
| Elementary school or below | Ref. |  | Ref. |  | |
| Middle school | 1.05 | (0.89, 1.25) | 0.74 * | (0.57, 0.97) | |
| Senior high school or technical school | 1.05 | (0.88, 1.26) | 0.73 * | (0.54, 0.97) | |
| Three-year college or associate degree | 1.04 | (0.85, 1.27) | 0.64 ** | (0.46, 0.88) | |
| Bachelor’s degree or above | 0.90 | (0.73, 1.10) | 0.54 ** | (0.38, 0.76) | |
| Quintiles of per capita monthly income |  |  |  |  | |
| Quintile 1 (CNY 0-1,000) | Ref. |  | Ref. |  | |
| Quintile 2 (CNY 1,001-1,600) | 0.95 | (0.83, 1.09) | 1.05 | (0.84, 1.32) | |
| Quintile 3 (CNY 1,601-2,400) | 0.93 | (0.81, 1.07) | 1.04 | (0.82, 1.31) | |
| Quintile 4 (CNY 2,401-3,750) | 0.92 | (0.80, 1.07) | 1.18 | (0.92, 1.52) | |
| Quintile 5 (CNY >3,751) | 0.95 | (0.81, 1.12) | 1.05 | (0.79, 1.40) | |
| Status of residence |  |  |  |  | |
| Local resident | Ref. |  | Ref. |  | |
| Inter-city migrant | 1.16 ** | (1.04, 1.29) | 1.09 | (0.90, 1.33) | |
| Place of residence |  |  |  |  | |
| Rural | Ref. |  | Ref. |  | |
| Urban | 0.96 | (0.87, 1.05) | 1.09 | (0.91, 1.29) | |
| DTaP, Diphtheria-tetanus-pertussis; CNY, Chinese Yuan, 1 CNY=0.14496 USD in 2019.  In the regressions, the province variable is controlled, and standard errors are clustered at the individual child level. RRR, relative-risk ratio; CI, confidence interval. ** p<0.01, * p<0.05. | | | | |  |

# **Supplementary Table 10. Association between the vaccine incident and NIP vaccine dose (except DTaP) delays by multinomial logistic regression (Base case: NIP doses not delayed)**

| Variables | NIP doses (except DTaP) delayed ≤3 months | | NIP doses (except DTaP) delayed >3 months | | |
| --- | --- | --- | --- | --- | --- |
|  | RRR | 95% CI | RRR | 95% CI | |
| Scheduled time for each dose |  |  |  |  | |
| Before vaccine incident | Ref. |  | Ref. |  | |
| After vaccine incident | 2.31 ** | (2.12, 2.52) | 4.31 ** | (3.77, 4.93) | |
| Child’s age (years) |  |  |  |  | |
| <1 | Ref. |  | Ref. |  | |
| 1-2 | 2.09 ** | (1.88, 2.32) | 7.83 ** | (5.97, 10.28) | |
| 2-3 | 3.83 ** | (3.38, 4.34) | 21.86 ** | (16.30, 29.31) | |
| 3-5 | 4.33 ** | (3.81, 4.93) | 27.75 ** | (20.37, 37.82) | |
| Child’s gender |  |  |  |  | |
| Female | Ref. |  | Ref. |  | |
| Male | 1.04 | (0.98, 1.11) | 1.14 * | (1.03, 1.27) | |
| Only one child in a family | 0.91 ** | (0.85, 0.98) | 0.72 ** | (0.64, 0.82) | |
| Guardian’s age (years) |  |  |  |  | |
| <30 | Ref. |  | Ref. |  | |
| 30-39 | 0.98 | (0.91, 1.06) | 1.04 | (0.91, 1.19) | |
| 40-49 | 0.98 | (0.86, 1.11) | 0.86 | (0.68, 1.07) | |
| ≥50 | 0.93 | (0.75, 1.16) | 0.89 | (0.57, 1.39) | |
| Guardian’s relationship with the child |  |  |  |  | |
| Mather | Ref. |  | Ref. |  | |
| Father | 1.05 | (0.97, 1.14) | 1.14 | (0.99, 1.31) | |
| Grandparent | 1.03 | (0.84, 1.28) | 0.95 | (0.62, 1.45) | |
| Ethnic groups |  |  |  |  | |
| Han | Ref. |  | Ref. |  | |
| Minorities | 0.93 | (0.81, 1.06) | 1.12 | (0.90, 1.41) | |
| Guardian’s education level |  |  |  |  | |
| Elementary school or below | Ref. |  | Ref. |  | |
| Middle school | 1.01 | (0.90, 1.13) | 1.04 | (0.83, 1.29) | |
| Senior high school or technical school | 1.01 | (0.89, 1.14) | 0.99 | (0.78, 1.27) | |
| Three-year college or associate degree | 1.02 | (0.89, 1.16) | 0.91 | (0.70, 1.18) | |
| Bachelor’s degree or above | 0.98 | (0.85, 1.12) | 0.86 | (0.66, 1.13) | |
| Quintiles of per capita monthly income |  |  |  |  | |
| Quintile 1 (CNY 0-1,000) | Ref. |  | Ref. |  | |
| Quintile 2 (CNY 1,001-1,600) | 0.93 | (0.85, 1.02) | 0.96 | (0.80, 1.15) | |
| Quintile 3 (CNY 1,601-2,400) | 0.91 * | (0.83, 1.00) | 0.96 | (0.81, 1.14) | |
| Quintile 4 (CNY 2,401-3,750) | 0.94 | (0.85, 1.04) | 1.04 | (0.87, 1.24) | |
| Quintile 5 (CNY >3,751) | 0.93 | (0.83, 1.04) | 0.96 | (0.79, 1.17) | |
| Status of residence |  |  |  |  | |
| Local resident | Ref. |  | Ref. |  | |
| Inter-city migrant | 1.09 * | (1.00, 1.17) | 1.07 | (0.94, 1.23) | |
| Place of residence |  |  |  |  | |
| Rural | Ref. |  | Ref. |  | |
| Urban | 1.08 * | (1.01, 1.16) | 1.17 * | (1.03, 1.33) | |
| DTaP, Diphtheria-tetanus-pertussis; NIP, National Immunization Program; CNY, Chinese Yuan, 1 CNY=0.14496 USD in 2019.  In the regressions, the province variable is controlled, and standard errors are clustered at the individual child level. RRR, relative-risk ratio; CI, confidence interval. ** p<0.01, * p<0.05. | | | | |  |

# **Supplementary Table 11. Association between the vaccine incident and non-NIP vaccine dose delays by multinomial logistic regression (Base case: non-NIP doses not delayed)**

| Variables | Non-NIP doses delayed ≤3 months | | Non-NIP doses delayed >3 months | | |
| --- | --- | --- | --- | --- | --- |
|  | RRR | 95% CI | RRR | 95% CI | |
| Scheduled time for each dose |  |  |  |  | |
| Before vaccine incident | Ref. |  | Ref. |  | |
| After vaccine incident | 0.92 | (0.76, 1.11) | 0.92 | (0.81, 1.05) | |
| Child’s age (years) |  |  |  |  | |
| <1 | Ref. |  | Ref. |  | |
| 1-2 | 0.87 | (0.68, 1.11) | 0.77 ** | (0.63, 0.94) | |
| 2-3 | 0.99 | (0.76, 1.30) | 1.09 | (0.88, 1.35) | |
| 3-5 | 0.93 | (0.70, 1.24) | 0.91 | (0.73, 1.14) | |
| Child’s gender |  |  |  |  | |
| Female | Ref. |  | Ref. |  | |
| Male | 1.15 * | (1.01, 1.32) | 1.23 ** | (1.09, 1.38) | |
| Only one child in a family | 0.96 | (0.82, 1.11) | 0.66 ** | (0.58, 0.75) | |
| Guardian’s age (years) |  |  |  |  | |
| <30 | Ref. |  | Ref. |  | |
| 30-39 | 0.95 | (0.80, 1.14) | 0.82 * | (0.71, 0.96) | |
| 40-49 | 0.92 | (0.70, 1.21) | 0.55 ** | (0.43, 0.70) | |
| ≥50 | 0.88 | (0.51, 1.53) | 0.52 ** | (0.33, 0.82) | |
| Guardian’s relationship with the child |  |  |  |  | |
| Mather | Ref. |  | Ref. |  | |
| Father | 0.99 | (0.81, 1.20) | 1.22 * | (1.03, 1.43) | |
| Grandparent | 0.86 | (0.52, 1.44) | 1.25 | (0.80, 1.94) | |
| Ethnic groups |  |  |  |  | |
| Han | Ref. |  | Ref. |  | |
| Minorities | 1.23 | (0.91, 1.66) | 1.21 | (0.93, 1.58) | |
| Guardian’s education level |  |  |  |  | |
| Elementary school or below | Ref. |  | Ref. |  | |
| Middle school | 1.00 | (0.75, 1.33) | 0.73 ** | (0.58, 0.92) | |
| Senior high school or technical school | 1.09 | (0.80, 1.49) | 0.85 | (0.65, 1.09) | |
| Three-year college or associate degree | 0.95 | (0.68, 1.33) | 0.67 ** | (0.51, 0.89) | |
| Bachelor’s degree or above | 0.80 | (0.58, 1.12) | 0.55 ** | (0.42, 0.73) | |
| Quintiles of per capita monthly income |  |  |  |  | |
| Quintile 1 (CNY 0-1,000) | Ref. |  | Ref. |  | |
| Quintile 2 (CNY 1,001-1,600) | 0.89 | (0.70, 1.13) | 0.75 ** | (0.62, 0.91) | |
| Quintile 3 (CNY 1,601-2,400) | 0.83 | (0.65, 1.06) | 0.67 ** | (0.56, 0.82) | |
| Quintile 4 (CNY 2,401-3,750) | 0.87 | (0.68, 1.12) | 0.65 ** | (0.53, 0.79) | |
| Quintile 5 (CNY >3,751) | 0.74 * | (0.57, 0.96) | 0.50 ** | (0.40, 0.62) | |
| Status of residence |  |  |  |  | |
| Local resident | Ref. |  | Ref. |  | |
| Inter-city migrant | 1.40 ** | (1.18, 1.67) | 1.27 ** | (1.08, 1.49) | |
| Place of residence |  |  |  |  | |
| Rural | Ref. |  | Ref. |  | |
| Urban | 0.70 ** | (0.60, 0.82) | 0.59 ** | (0.51, 0.67) | |
| NIP, National Immunization Program; CNY, Chinese Yuan, 1 CNY=0.14496 USD in 2019.  In the regressions, the province variable is controlled, and standard errors are clustered at the individual child level. RRR, relative-risk ratio; CI, confidence interval. ** p<0.01, * p<0.05. | | | | |  |
